# Gamma-band auditory steady-state response after frontal tDCS: A double-blind, randomized, crossover study

**DOI:** 10.1371/journal.pone.0193422

**Published:** 2018-02-28

**Authors:** Yoshiaki Miyagishi, Takashi Ikeda, Tetsuya Takahashi, Kiwamu Kudo, Hirofumi Morise, Yoshio Minabe, Mitsuru Kikuchi

**Affiliations:** 1 Department of Psychiatry and Neurobiology, Graduate School of Medical Science, Kanazawa University, Kanazawa, Japan; 2 Research Center for Child Mental Development, Kanazawa University, Kanazawa, Japan; 3 Health Administration Center, University of Fukui, Fukui, Japan; 4 Ricoh Institute of Future Technology, Research and Development Division, Ricoh Company, Ltd., Kanazawa, Japan; Hamamatsu University School of Medicine, JAPAN

## Abstract

The effects of transcranial direct current stimulation (tDCS) likely depend on cortical N-methyl-D-aspartic acid (NMDA) neurotransmission; however, no previous studies have reported tDCS-mediated modulation of cortical NMDA neurotransmission in humans. The gamma-band auditory steady-state response (ASSR) to a 40 Hz stimulation likely reflects the integrity of cortical NMDA neurotransmission. The present study tested whether the effect of tDCS is reflected in gamma-band ASSRs during a 40 Hz stimulation. Using a double-blind, randomized, crossover study, we performed magnetoencephalography (MEG) and measured the ASSR in 24 healthy participants during 40 Hz of auditory stimulation after prefrontal tDCS (2 mA) or sham (i.e., placebo) treatment. Our results failed to reveal significant differences in any brain between the two conditions after the application of a frequency of approximately 40 Hz. Based on these results, the ASSR is an insufficient method to detect the effect of tDCS on cortical NMDA neurotransmission. Unexpectedly, the results revealed an enhanced beta-band event-related spectral perturbation (ERSP) in the left motor cortex after tDCS compared with that observed after the sham stimuli. Given that beta-band oscillations reflect many functions in motor cortices, the tDCS for the frontal areas had some effect on the left motor cortex while the participants were focusing on not pressing the button with their right index finger. An additional study with an adequate psychological task is necessary to draw a conclusion regarding this unexpected result.

## Introduction

Transcranial direct current stimulation (tDCS) is a form of electrical neuro-stimulation [1]. To date, tDCS has been used to treat a variety of psychiatric (e.g., major depressive disorder and schizophrenia) and neurological conditions [2–5], and it has been used to alter performance on a range of cognitive tasks [6, 7]. According to the results of an animal study, anodal stimulation applied directly to the cortex increases the positive character of the resting membrane potential, whereas cathodal stimulation causes hyperpolarization [8]. Previous neuroimaging studies of humans revealed that anodal stimulation tends to increase the blood oxygen level-dependent (BOLD) signal, whereas cathodal stimulation decreases this signal [9, 10]. Intriguingly, many previous studies have reported the effectiveness of left dorsolateral prefrontal anodal tDCS as a treatment for major depressive disorder [4, 5] and schizophrenia [11], as well as working memory performance in healthy subjects [7].

tDCS is associated with the modulation of glutamatergic, gamma-aminobutyric acidergic (GABAergic), dopaminergic, serotonergic, and cholinergic activities [12]. These modulations likely affect plasticity processes, making tDCS an important tool for clinical treatment and for understanding cognitive ability. Notably, previous pharmacological studies using transcranial magnetic stimulation revealed that the effects of tDCS depend on cortical N-methyl-D-aspartic acid (NMDA) neurotransmission and the GABA_A_ receptor [13, 14]; however, no previous studies have investigated whether tDCS of the frontal area modulates cortical NMDA neurotransmission in humans.

The auditory steady-state response (ASSR) is an electro-magneto-physiological response entrained to both the frequency and phase of rapid and periodic auditory stimuli [15] and is typically captured using noninvasive techniques such as electroencephalography (EEG) and magnetoencephalography (MEG). This response is most evident in humans when stimuli are presented in the gamma frequency range (30–50 Hz) [15]. Notably, the ASSR in the gamma frequency range is likely the most robust finding of abnormal gamma oscillations recorded by either EEG [16–23] or MEG [24–28] in patients with schizophrenia [29]. In addition, the ASSR recorded by EEG during 40 Hz of stimulation likely reflects the integrity of cortical NMDA neurotransmission [30, 31].

MEG has been in development for human use since the 1960s, and its development has been greatly aided by EEG studies and recent advances in computational algorithms and hardware [32]. EEG-sensing technology is extremely mature (over 80 years of history) and relatively cost effective compared with other neuroimaging devices. MEG has a relatively short history and is generally very expensive. However, evidence from healthy populations (comparing the sensitivity of EEG to MEG for the measurement of gamma-band oscillations) has highlighted the improved detectability of high-frequency activity (i.e., gamma-band oscillation) using MEG measurements [33]. Recent evidence suggests that an MEG-informed reconstruction of the source significantly enhances the signal-to-noise ratio of 40-Hz ASSR estimates during normal brain functioning [34]. To our knowledge, however, no previous MEG studies have investigated the effect of tDCS on gamma-band ASSRs in humans. The present study tested whether gamma-band ASSRs are altered after tDCS (left frontal anodal tDCS).

## Materials and methods

### Participants

Twenty-four right-handed adult male students (mean age = 21.3, range = 20–23 years old) were recruited from Kanazawa University and participated in this experiment. All participants were native Japanese speakers with normal hearing and had no medical or family histories of neurological or psychiatric disorders. The full IQ score was estimated using the Japanese version of the National Adult Reading Test (mean = 108.4, range 93–120) [35]. These men agreed to participate in this study with full knowledge of the experimental nature of the research. Each participant provided written informed consent prior to participation. The Ethics Committee of Kanazawa University approved this study, which conformed to the tenets of the Declaration of Helsinki.

### Experimental design

**Fig 1:**
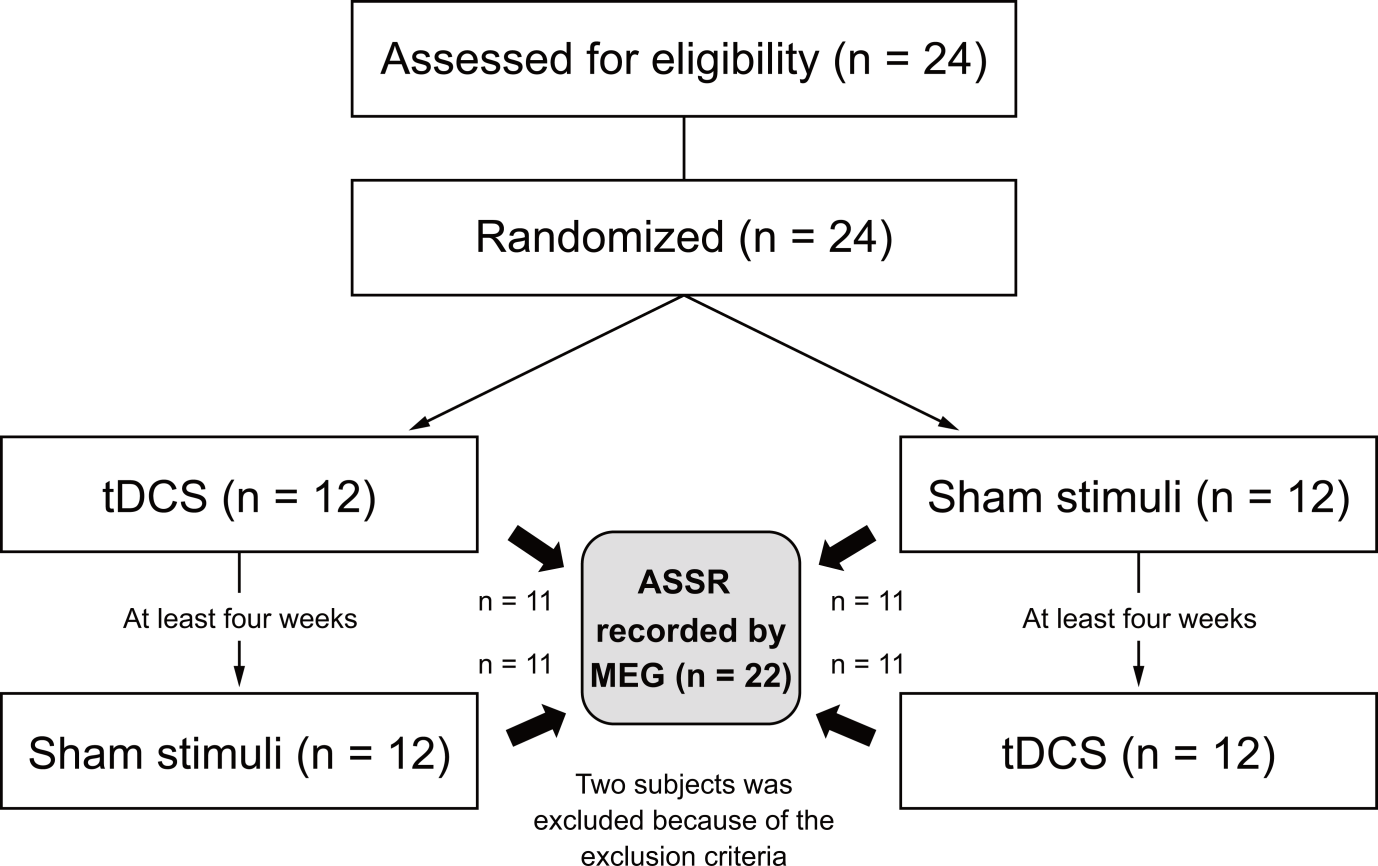
Study design: A double-blind, randomized, crossover study. Twenty-four participants were recruited and randomly assigned to receive either tDCS or sham stimulation during the first trial. After an interval of at least four weeks, the second trial was conducted. The order of the two stimulation conditions (i.e., tDCS or sham) was counterbalanced across participants. After delivering one of the two stimulus conditions, ASSRs were recorded using MEG. We excluded two participants from the statistical analysis because they met the exclusion criteria. ASSR, auditory steady-state response.

The study employed a randomized double-blind controlled placebo (active tDCS, sham) crossover design that included an interval of at least one month (mean interval = 57.4 days). At the beginning of the experiment, participants were randomly assigned to either the active tDCS or sham stimulation condition. Ten minutes (the preparation time for MEG measurement) after stimulation, we measured the neuromagnetic ASSR to investigate the effect of tDCS (Fig 1).

### tDCS

**Fig 2:**
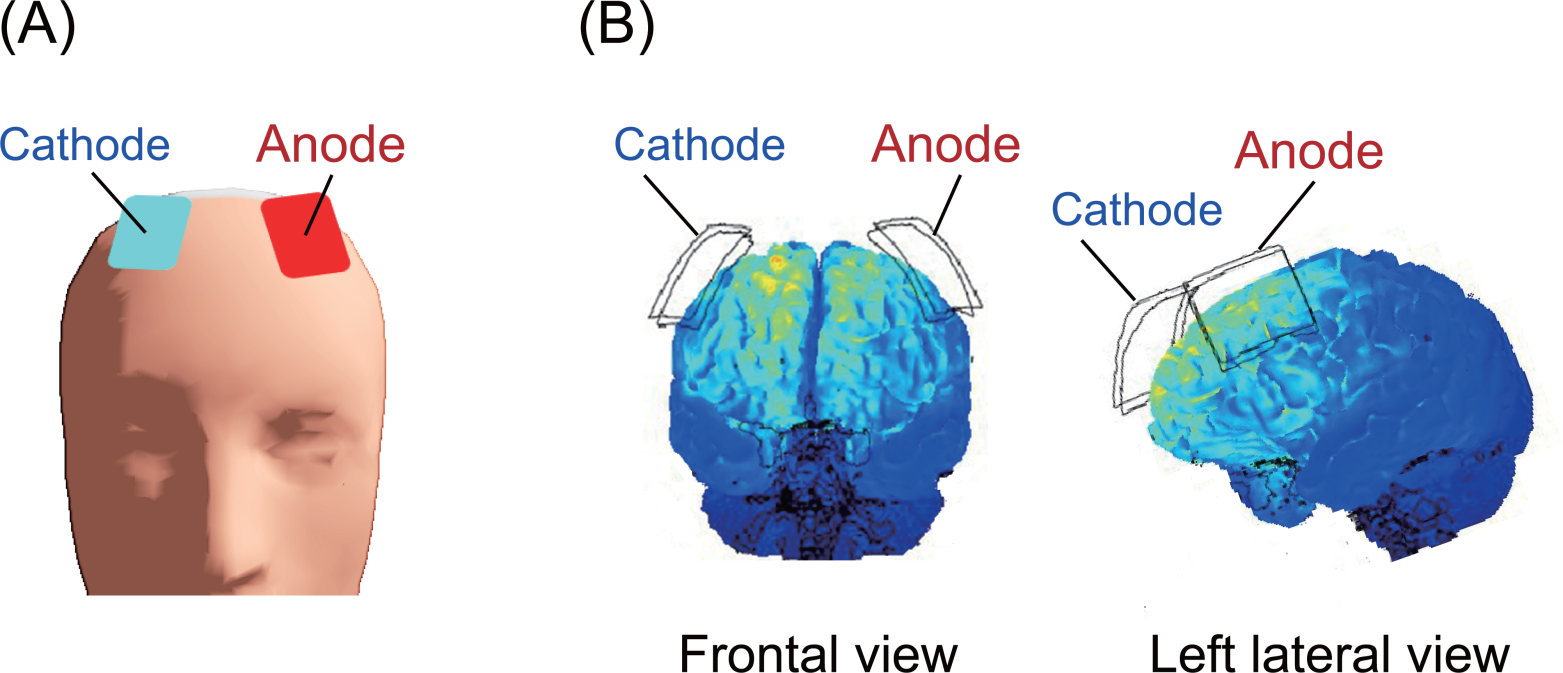
Electrode placement. (A) Anodal (F3) and cathodal (F4) electrodes marked on the scalp surface. (B) The computational simulation of brain current flow during the delivery of the tDCS. Major changes in the brain current flow were observed in the dorsal-frontal areas during stimulation [37].

A direct current was induced through two saline-soaked surface sponge electrodes (35 cm^2^) and delivered using a battery-driven, constant current stimulator (DC-STIMULATOR Plus, neuroConn GmbH, Germany). The anode electrode was placed over F3, and the cathode electrode was placed over F4 (see the international EEG 10/10 system) during stimulation (Fig 2). Participants received the stimulus twice, and the duration of the stimulation was 13 minutes at a current strength of 2 mA to maximize the subsequent effects of stimulation. Twenty minute inter-stimulation intervals were employed between stimuli [36]. During the sham stimulation, electrodes were also attached to the participant, but the current was only delivered during the first 10 seconds, which prevented the participants from noticing the absence of electrical stimulation.

### ASSR

The ASSR session included passive listening tasks in which 250 trials of click-train stimuli were presented binaurally at 80 dB sound pressure level for 1000 ms each, with an inter-trial interval of 2000 ms. The click-train stimulus was a series of 1 kHz single sine-wave stimuli administered at a stimulation frequency of 40 Hz. The stimuli were received using stereo earphones with earplugs (ER-30, Etymotic research Inc., IL, USA). During the scan, participants were instructed to look at a white fixation cross on a black background presented on the screen in front of them. Participants were required to perform one task to confirm their awake state. Their task was to detect the 2 kHz click-train stimulus presented ten times during the session (the button press cue proportion was 4%). When the 2 kHz stimulus was presented, participants pressed a button (LUMINA LU400-PAIR, Cedrus Corporation, CA, USA) with their right index fingers. All stimuli were controlled using Presentation (Version 13.1, Neurobehavioral Systems, CA, USA) for Windows XP. A trigger signal was sent to an MEG recording system to indicate the start of the sound stimulus.

### Data acquisition

Magnetic fields were measured using a whole-head-type system for adults at a laboratory within Ricoh Company in Japan. This system (MEGvision PQA160C; Ricoh Company, Ltd., Kanazawa, Japan) consisted of 160 channels. Sensors were configured as first-order coaxial gradiometers with a baseline of 50 mm; each coil of the gradiometers measured 15.5 mm in diameter. Magnetic fields were sampled at 2000 Hz per channel with a 500 Hz low-pass filter. Using a Signa Excite HD 1.5 T system (GE Yokogawa Medical Systems Ltd., Milwaukee, WI, USA), we obtained a T1-weighted structural image with spherical lipid markers placed at the 5 MEG fiduciary points to enable us to superpose the MEG coordinate system on the MRI data. A T1-weighted image consisted of 166 sequential 1.2 mm-thick slices with a resolution of 512 × 512 points within a field of view of 261 × 261 mm. The cortex surface was reconstructed using Freesurfer (version 5.3, http://surfer.nmr.mgh.harvard.edu/).

### MEG data analyses and statistics

All data processing and analytical procedures were performed using Brainstorm [38], and additional scripts were developed using MATLAB® (The MathWorks, Natick, MA, USA). Noisy or flat channels were eliminated from the analysis. Eye-movement and cardiac artifacts were removed using the signal-space projection (SSP) method. Segments that included head movement or muscle artifacts detected in a visual inspection or the automatic processing procedure in Brainstorm were discarded. The epoch was defined as -500 to 1500 ms relative to the auditory stimulus onset (0 ms). Data were then baseline-corrected with respect to the mean of the prestimulus period (from -500 to 0 ms).

We estimated the signal source using the anatomy of each subject. The lead field was then computed using the overlapping spheres algorithm [39], with a cortical surface tessellated with 15,000 vertices. The inverse solution was calculated for each individual using sLORETA [40]. A noise covariance matrix was calculated based on the MEG recordings obtained during the -500 to 0 ms time window of every epoch within a session. Regions of interest (ROIs) were determined based on the Desikan-Killiany gyrus atlas segmented by Freesurfer.

A time-frequency analysis was conducted using Morlet wavelets with a central frequency of 2 Hz; a time resolution of 3 s was chosen for the mother wavelet. We calculated the event-related spectral perturbation (ERSP) and inter-trial phase coherence (ITPC) from 2 to 50 Hz. The ERSP represents the event-related percent changes in signal magnitude relative to a prestimulus baseline period (from -200 to 0 ms). The ITPC was calculated using complex values based on the raw output from the time-frequency analysis function of Brainstorm, and it indicates the phase consistency across trials. The time-frequency maps of all ROIs are shown in supporting information S1 Fig (ERSP) and S2 Fig (ITPC).

### Statistics

To compare the neural activation under the tDCS and sham conditions, we conducted paired *t*-tests (21 degrees of freedom) for each ROI within each time frame from 0 to 1500 ms and for each frequency frame from 2 to 50 Hz. The threshold for statistical significance was set to *p* < 0.05, with a false discovery rate (FDR) correction across all 68 ROIs and 49 frequency dimensions. The same procedure was applied to the analyses of ERSP and ITPC data.

## Results

Two of the 24 participants were excluded. One participant was excluded from further analysis because of drowsiness during the MEG recording. The other participant was excluded because of magnetic contamination from a tooth filling. Ultimately, twenty-two participants completed all sessions (Fig 1). The mean estimated IQ score in the twenty-two participants was 107.7 (range 93–120).

### ERSP in the 40 Hz ASSR

**Fig 3:**
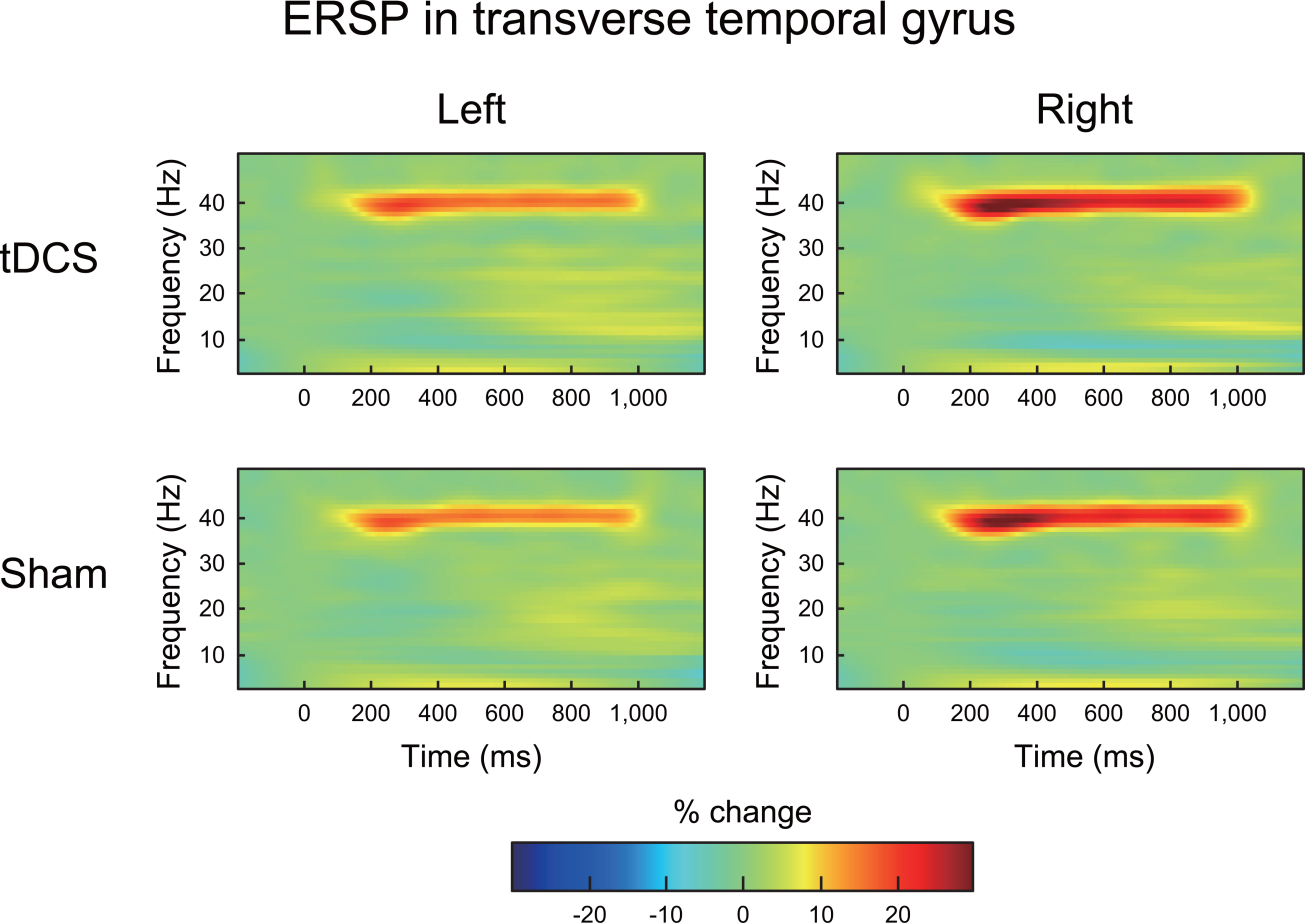
The grand average of the time-frequency maps of the ERSP in the transverse temporal gyrus under both tDCS and sham conditions. In each map, the x-axis indicates time (ms), and the y-axis indicates frequency (Hz). The color indicates the ERSP at each time-frequency point (reflected as the percentage change from baseline). The gamma-band (40 Hz) ASSR derived from the 40 Hz auditory stimulation was clearly observed under the tDCS and sham conditions.

**Fig 4:**
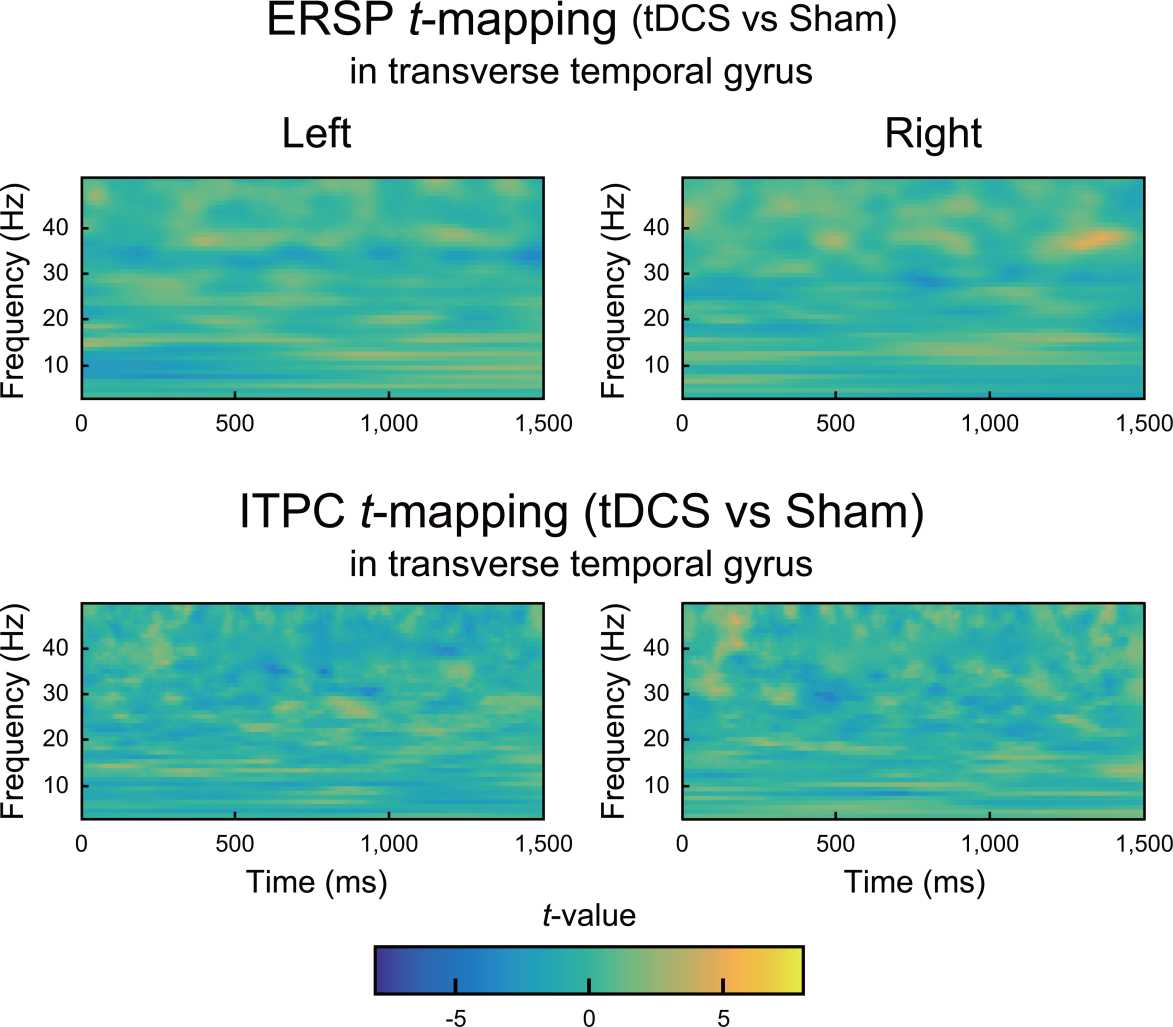
*T*-maps of the differences in ERSPs (upper row) and ITPCs (lower row) in the transverse temporal gyrus between the tDCS and sham conditions. Color indicates *t*-values at each time-frequency point. No significant differences were found in either ERSPs or ITPCs when the statistical threshold was set to *p* < 0.05 with an FDR correction.

An ERSP at approximately 40 Hz was clearly observed in most ROIs during 40 Hz of auditory stimulation under both the tDCS and sham conditions. A representative ERSP at approximately 40 Hz is shown in the transverse temporal gyrus in Fig 3. Paired-sample t-tests failed to identify significant differences in ERSPs at approximately 40 Hz in any of the ROIs when the statistical threshold was set to *p* < 0.05 with FDR corrections across ROIs and frequency dimensions (Fig 4; upper row).

### ITPC in the 40 Hz ASSR

**Fig 5:**
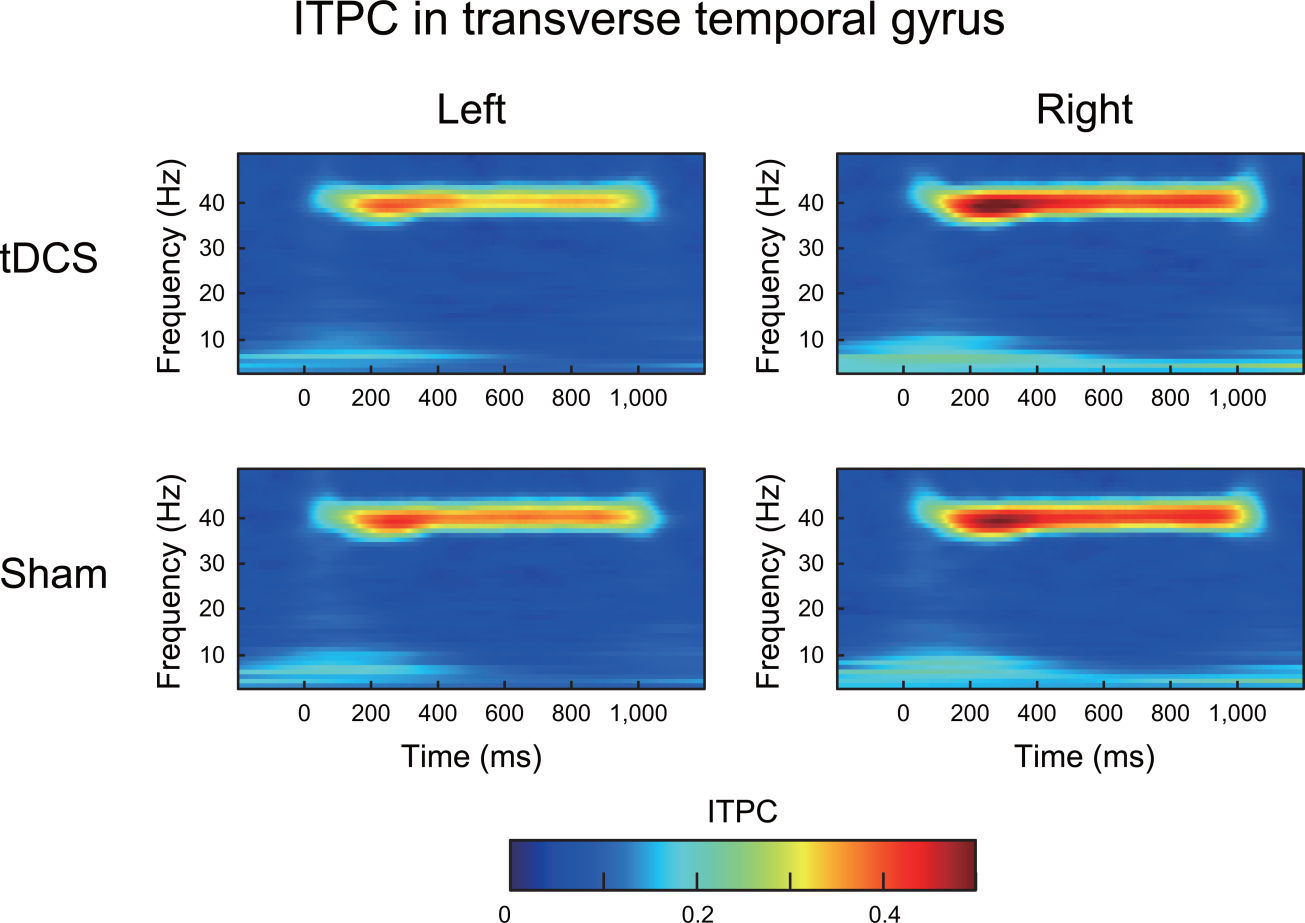
The grand average of the time-frequency maps for the ITPC in the transverse temporal gyrus under both tDCS and sham conditions. In each map, the x-axis indicates time (ms), and the y-axis indicates frequency (Hz). The color indicates the ITPCs at each time-frequency point. The ITPC peak in the gamma-band (40 Hz) was clearly observed during the 40 Hz auditory stimulation.

An ITPC at approximately 40 Hz was clearly observed in most of the ROIs during the 40 Hz auditory stimulation under both the tDCS and sham conditions. A representative ERSP at approximately 40 Hz is shown in the transverse temporal gyrus in Fig 5. Paired-sample t-tests failed to identify significant differences in ERSPs at approximately 40 Hz in any of the ROIs when the statistical threshold was set to *p* < 0.05 with FDR corrections across ROIs and frequency dimensions (Fig 4; lower row).

### ERSPs and ITPCs in other bands

**Fig 6:**
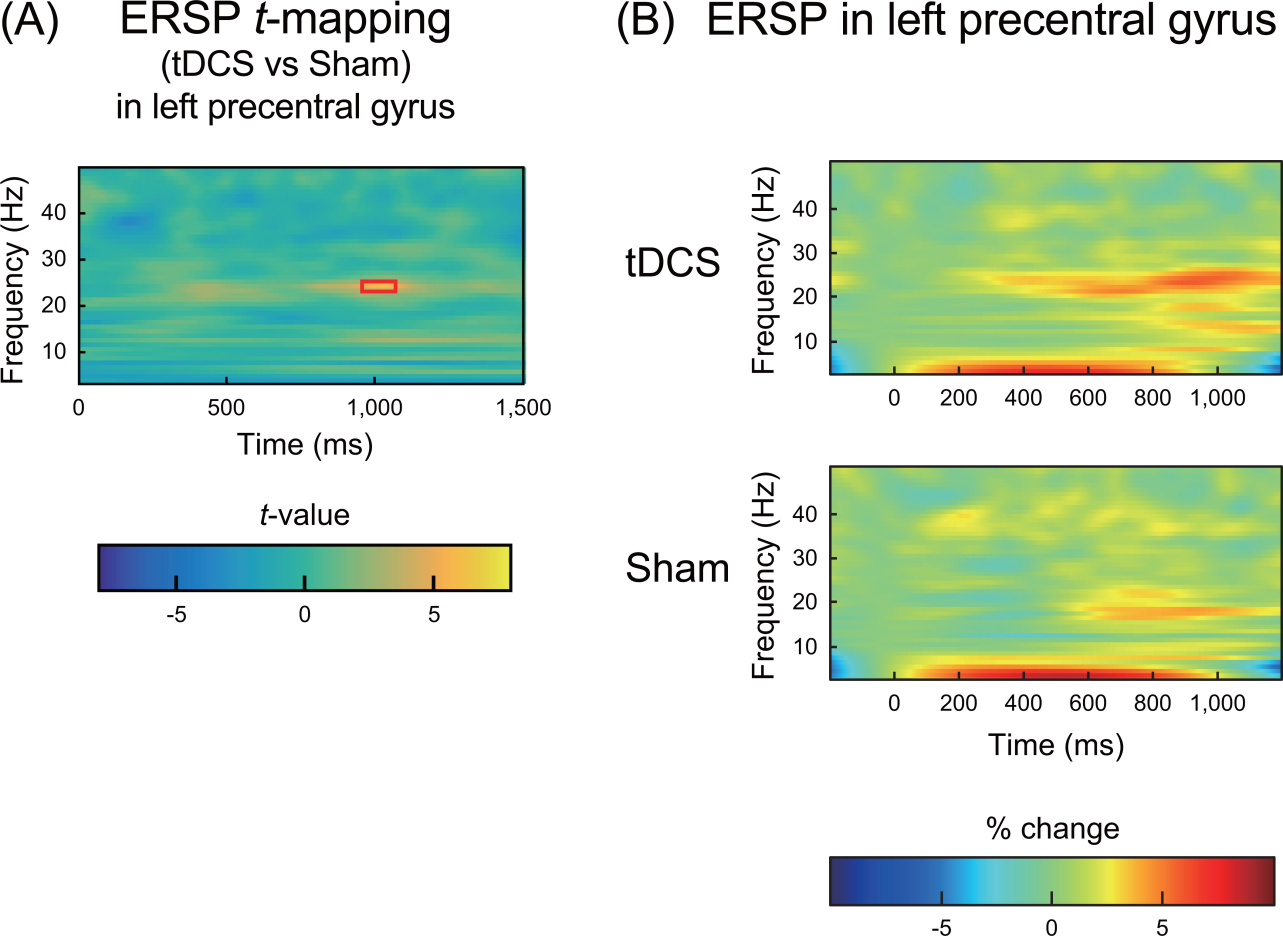
ERSPs in the left precentral gyrus. (A) *T*-maps of the differences in the ERSPs between the tDCS and sham conditions. The threshold of *p* < 0.05 (FDR corrected) is highlighted with a red rectangle. The color indicates the *t*-values at each time-frequency point. Significantly higher ERSPs were observed in the beta-band (20 Hz; a red rectangle) after tDCS than after the sham condition. (B) The grand average of the time-frequency maps of ERSPs in the left precentral gyrus under both tDCS (upper row) and sham (lower row) conditions. In each map, the x-axis indicates time (ms), and the y-axis indicates frequency (Hz). The color indicates ERSPs at each time-frequency point (reflected as the percentage change from baseline).

Outside of the gamma-band range, paired-samples t-tests unexpectedly revealed an enhanced beta-band ERSP in the left motor cortex (precentral gyrus) after tDCS compared with that observed after the sham stimuli (Fig 6A; highlighted with a red rectangle). Fig 6B shows the ERSP in the left precentral gyrus. No significant differences in ITPCs were observed in any bands for any ROIs.

## Discussion

To our knowledge, this study is the first to investigate the effects of frontal tDCS on gamma-band ASSRs in humans. However, we failed to observe a significant effect of tDCS (left frontal anodal tDCS) on either ERSPs or ITPCs in the gamma-band for any ROI. Based on these results, a 40 Hz ASSR is insufficient to detect the effect of tDCS on cortical NMDA neurotransmission. One possible explanation might include the lack of statistical power needed to detect the subtle effect of tDCS on cortical NMDA neurotransmission. In addition, the gamma-band ASSR used in the present study might have been inadequate to detect subtle changes in NMDA neurotransmission induced by tDCS. Alternatively, tDCS might not have a specific effect on cortical NMDA neurotransmission, although this hypothesis is inconsistent with the results of previous pharmacological studies [13].

Unexpectedly and intriguingly, we observed an enhanced beta-band ERSP in the left motor cortex after tDCS compared with that observed after the sham stimuli. This enhanced beta-band ERSP in the left motor cortex (precentral gyrus) might reflect the inhibition of cortical activity by tDCS. Go/No-Go paradigms consist of a series of stimulus-induced movements in which the subject must decide whether to move depending on the characteristics of the stimulus. These paradigms have been extensively used to study processes linked to decision making and response inhibition. Usually, the behavioral index of inhibitory control is the number of errors a subject makes on the No-Go trials (i.e., false alarms [FAs]). In the present study, participants were required to detect the deviant click-train stimulus presented ten times in a session. When the deviant stimulus was presented, participants were required to press a button with their right index fingers (the proportion of Go tasks was 4%), and they were required to fail to respond to the standard stimulus (the proportion of No-Go tasks was 96%). Therefore, we might regard our ASSR session as an atypical Go/No-Go task, and the enhanced beta-band ERSP in the left motor cortex might reflect the No-Go task-related cortical activity modulated by tDCS. However, the behavioral results did not reveal an effect of tDCS on the Go/No-Go performance. In the present study, the FA means (range) per stimulation were 0.20 (0–0.8) and 0.38 (0–1.6) for the tDCS and sham conditions, respectively, and no significant differences in the number of FAs were observed between the two conditions (Wilcoxon signed-rank test, *z* = 1.34, *p* > 0.05).

Previous studies have focused on the EEG event-related desynchronization (ERD = decreased ERSP) and synchronization (ERS = increased ERSP) in the beta-band during Go/No-Go paradigms [41, 42]. In these studies, No-Go trials displayed an increase in beta power (i.e., ERS) [41, 42]. In addition, one previous study used intracranial recordings and reported that the beta power over the inferior frontal areas increased relative to the baseline during inhibited stop trials but not during failed inhibitions [43]. Therefore, beta-band-enhanced ERSPs likely reflect frontal inhibitory processes. The present study unexpectedly revealed enhanced beta-band ERSPs in the left motor cortex after tDCS compared with that observed after the sham stimuli (Fig 6A; highlighted with a red rectangle). Although the behavioral results in the present study did not reveal an effect of tDCS on the Go/No-Go performance, tDCS with the anode electrode placed over the left frontal area and the cathode electrode placed over the right frontal area might have had an effect on the frontal inhibitory system while the participants were focusing on not pressing the button with their right index finger.

Regarding the tDCS results during the inhibitory task, two previous studies reported the effect of prefrontal tDCS (1.5 mA). Cathodal stimulation of the right dorsolateral prefrontal cortex (DLPFC) increased FAs in healthy subjects during a Go/No-Go task in one study [44]. In another study, cathodal stimulation of the left DLPFC decreased FAs in high school students with attention-deficit/hyperactivity disorder (ADHD) during a Go/No-Go task [45]. Therefore, based on the results from the present study, tDCS applied with the anode electrode placed over the left frontal area and the cathode electrode placed over the right frontal area diminishes the frontal inhibitory processes during Go/No-Go paradigms. However, the results from the present study did not show this effect, i.e., the mean FA per trial was lower under the tDCS conditions (0.20) than under the sham condition (0.38; Wilcoxon signed-rank test, *z* = 1.34, *p* > 0.05, not significant). This inconsistency might be partially explained by the differences in stimulus intensity. Previous studies used lower intensity (1.5 mA) stimuli, whereas we used higher intensity (2 mA) stimuli. One previous study of the motor cortex reported that low-intensity (1 mA) stimulation causes the conventional polarity-specific modulation of neural excitability, whereas high-intensity (2 mA) stimulation leads to increased excitability from both stimulation polarities [46]. In addition to stimulus intensity, the reliability of the induced excitability changes can vary from session to session within individuals and across participants [47]. Because of these uncertain factors in the present study, we might not have observed a significant effect on behavioral performance (i.e., FAs).

## Conclusions

In conclusion, the ASSR is an insufficient method to detect the effect of tDCS on cortical NMDA neurotransmission. We unexpectedly observed enhanced beta-band ERSPs in the left motor cortex after tDCS of the frontal area compared with that observed after the sham stimuli. Given that beta-band oscillations reflect many functions in motor cortices, the tDCS for the frontal areas had some effect on the left motor cortex while the participants were focusing on not pressing the button with their right index finger. Further studies with an adequate psychological task (i.e., adequate Go/No-Go proportions) and tDCS of varying intensity and location are necessary to draw conclusions about this unexpected result.

## Supporting information

S1 Fig The grand average of the time-frequency maps for the ERSP in all the ROIs under both tDCS and sham conditions. In each map, the x-axis indicates time (ms), and the y-axis indicates frequency (Hz). The color indicates the ITPCs at each time-frequency point. The ITPC peak in the gamma-band (40 Hz) was clearly observed during the 40 Hz auditory stimulation.
(PDF)

S2 Fig The grand average of the time-frequency maps for the ITPC in all the ROIs under both tDCS and sham conditions. In each map, the x-axis indicates time (ms), and the y-axis indicates frequency (Hz). The color indicates the ITPCs at each time-frequency point. The ITPC peak in the gamma-band (40 Hz) was clearly observed during the 40 Hz auditory stimulation.
(PDF)
