# Supplementary material for: Gamma-band auditory steady-state response after frontal tDCS: A double-blind, randomized, crossover study
Source: PLoS One. 2018 Feb 28;13(2):e0193422. doi: 10.1371/journal.pone.0193422 (PMC5830999; doi:10.1371/journal.pone.0193422)

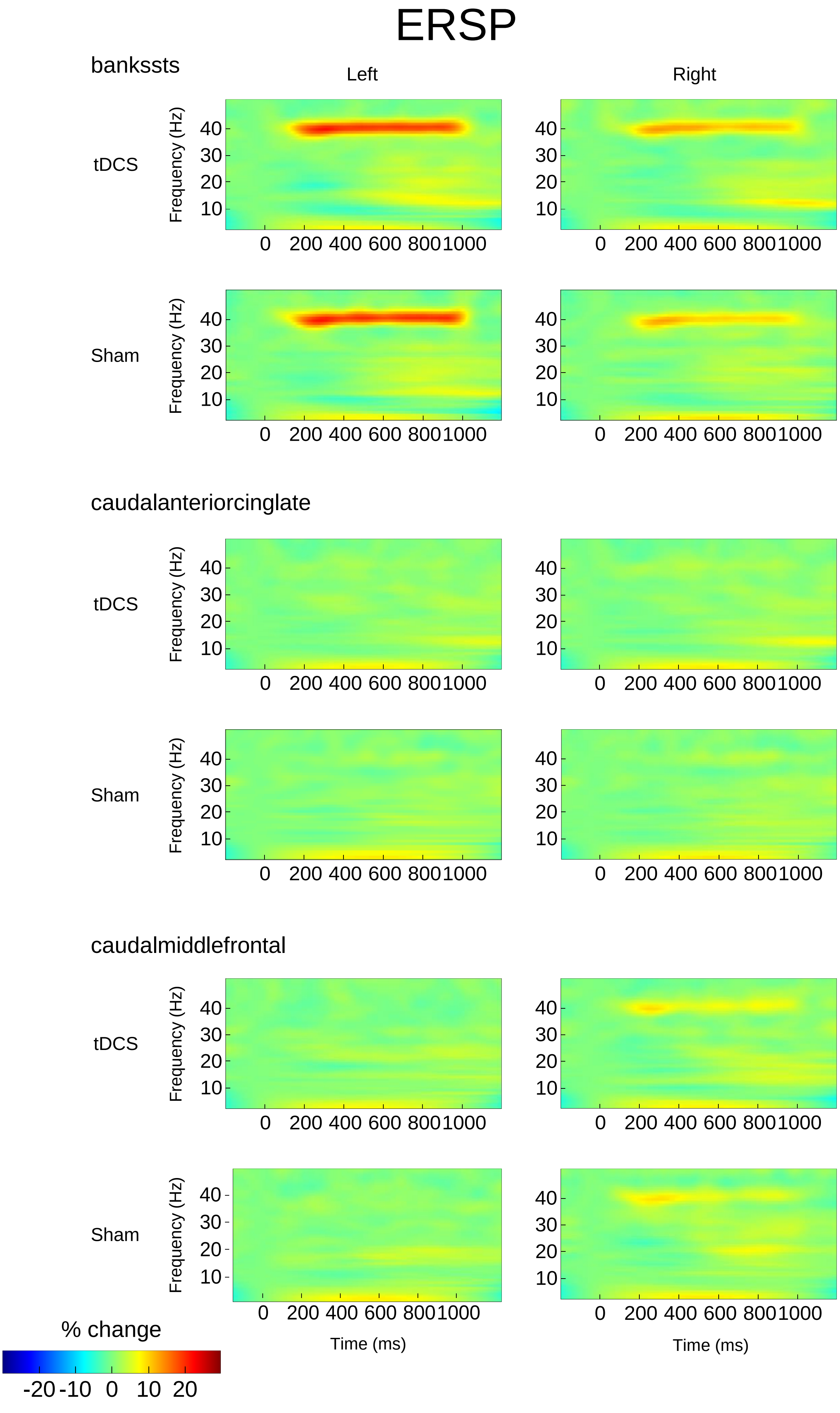
ERSP
bankssts
Left
Right
tDCS
Sham
Frequency (Hz)
40
30
20
10
0 200 400 600 800 1000
caudalanteriorcinglate
caudalmiddlefrontal
Time (ms)
% change
-20 -10 0 10 20

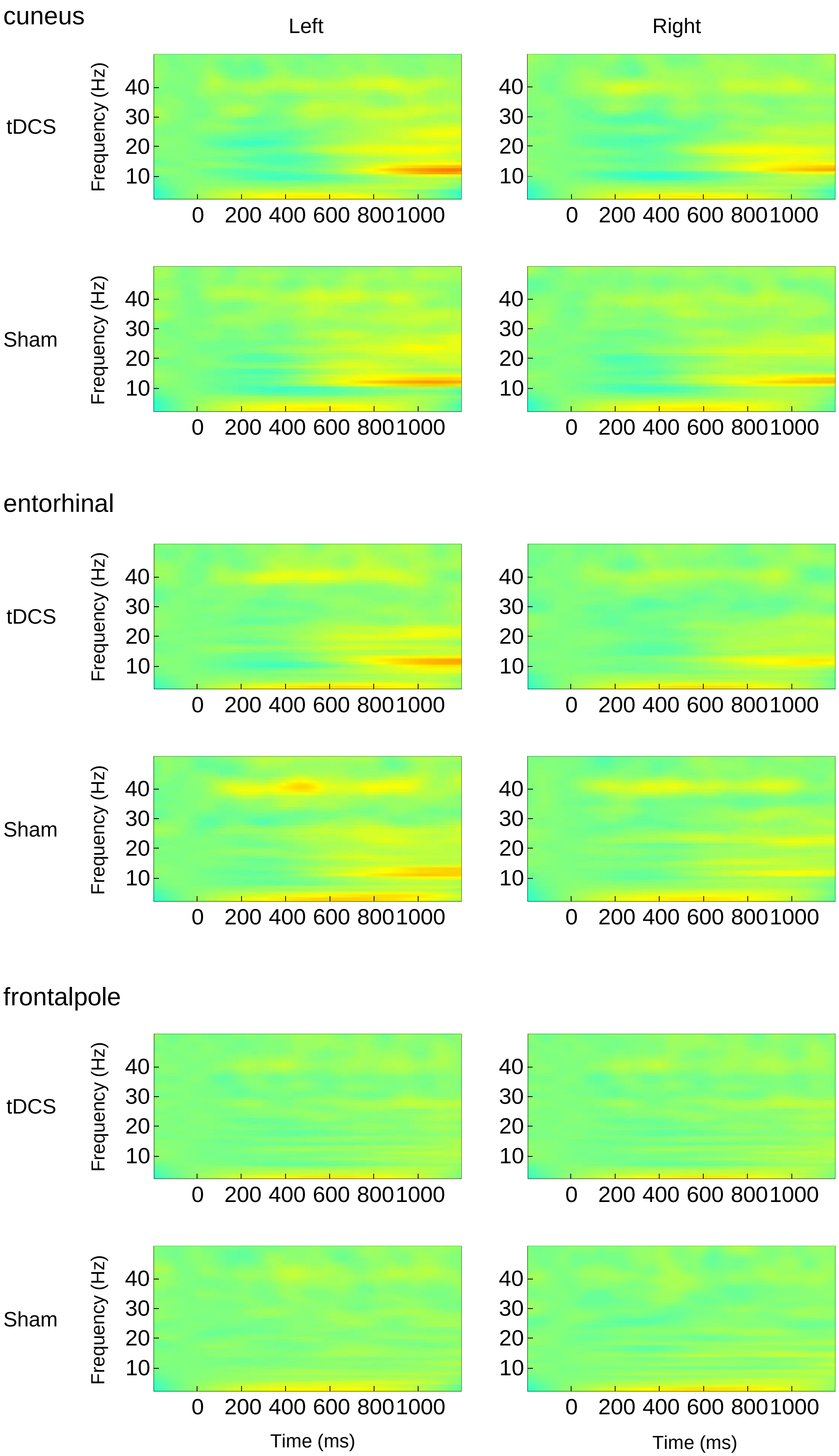

cuneus
Left
Right
tDCS
Sham
entorhinal
frontalpole
Frequency (Hz)
10
20
30
40
0
200
400
600
800
1000
Time (ms)

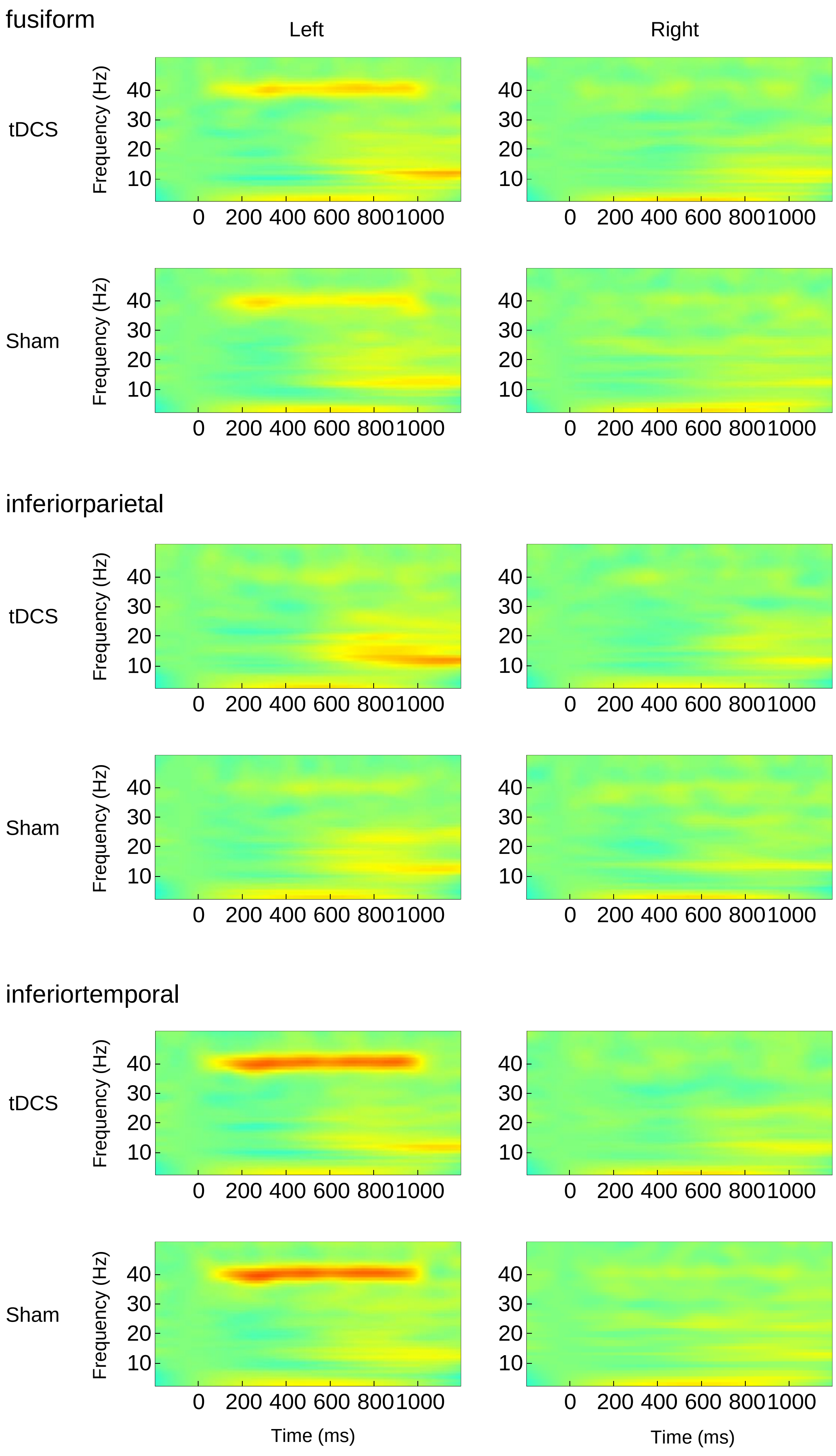
fusiform
Left
Right
tDCS
Sham
inferiorparietal
tDCS
Sham
inferiortemporal
tDCS
Sham
Frequency (Hz)
40
30
20
10
0 200 400 600 800 1000
Time (ms)
Time (ms)

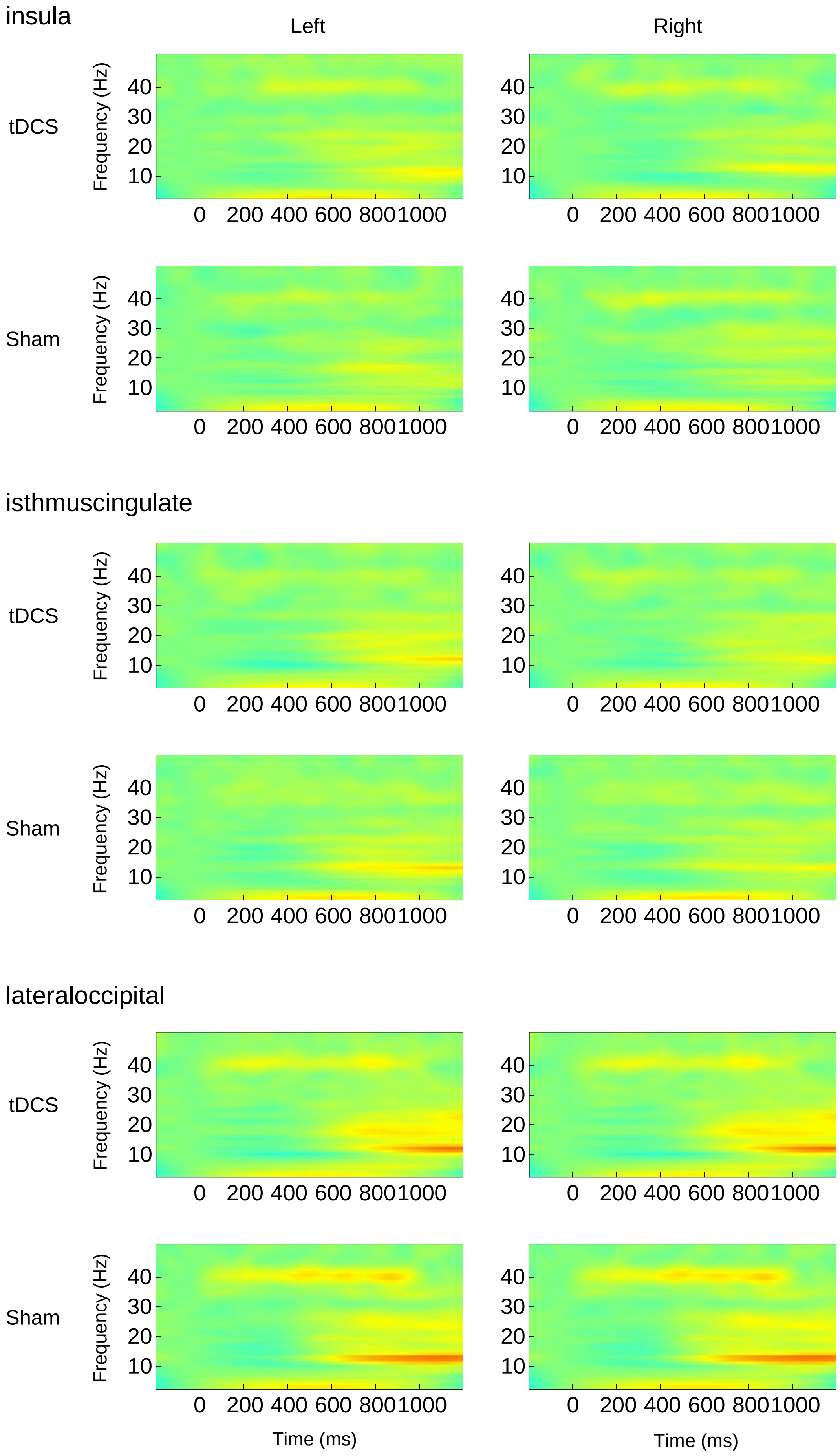
insula
Left
Right
tDCS
Sham
Frequency (Hz)
40
30
20
10
0
200
400
600
800
1000
isthmuscingulate
lateraloccipital
Time (ms)

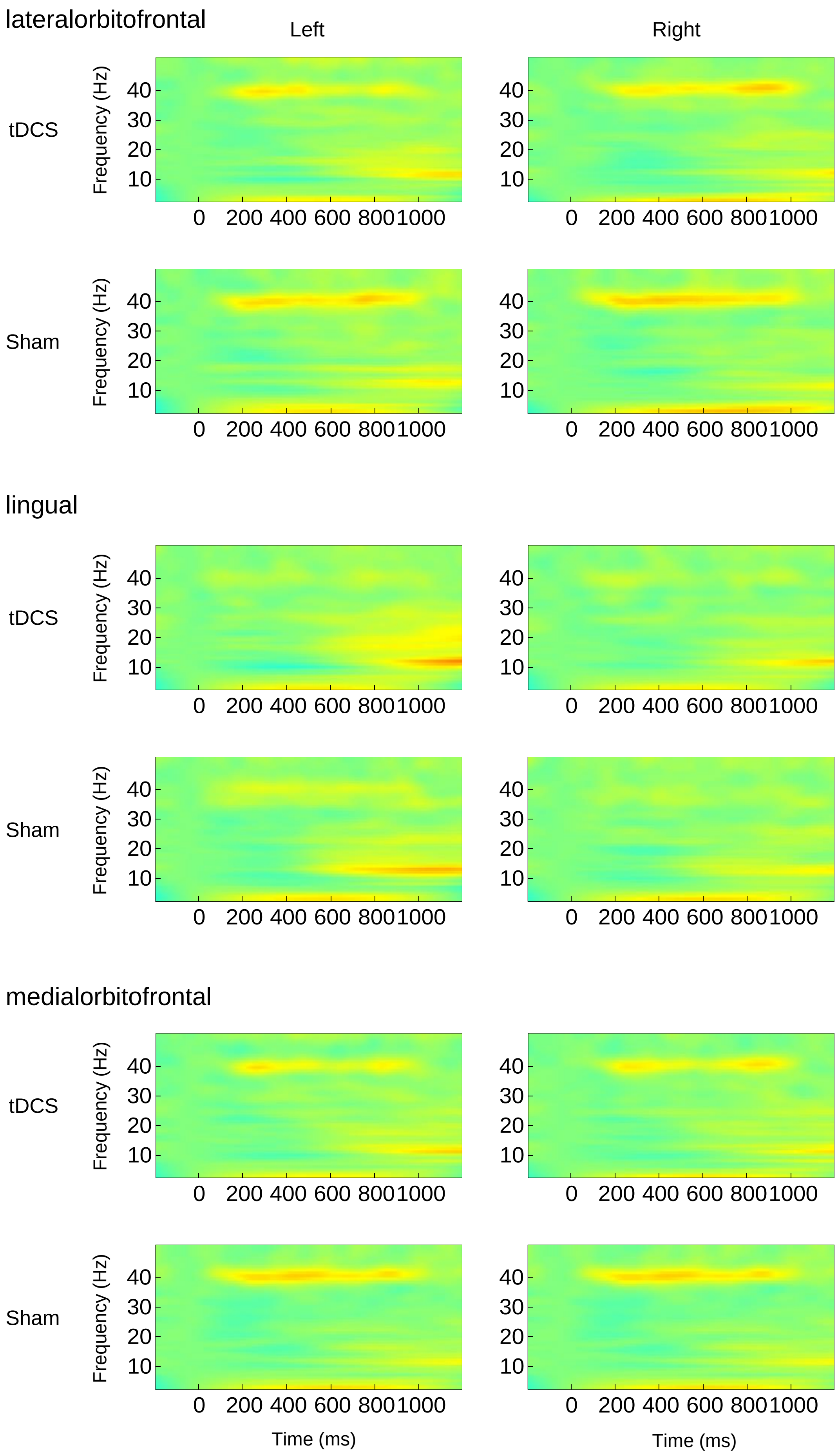
lateralorbitofrontal
Left
Right
tDCS
Sham
lingual
tDCS
Sham
medialorbitofrontal
tDCS
Sham
Frequency (Hz)
40
30
20
10
0
200
400
600
800
1000
Time (ms)

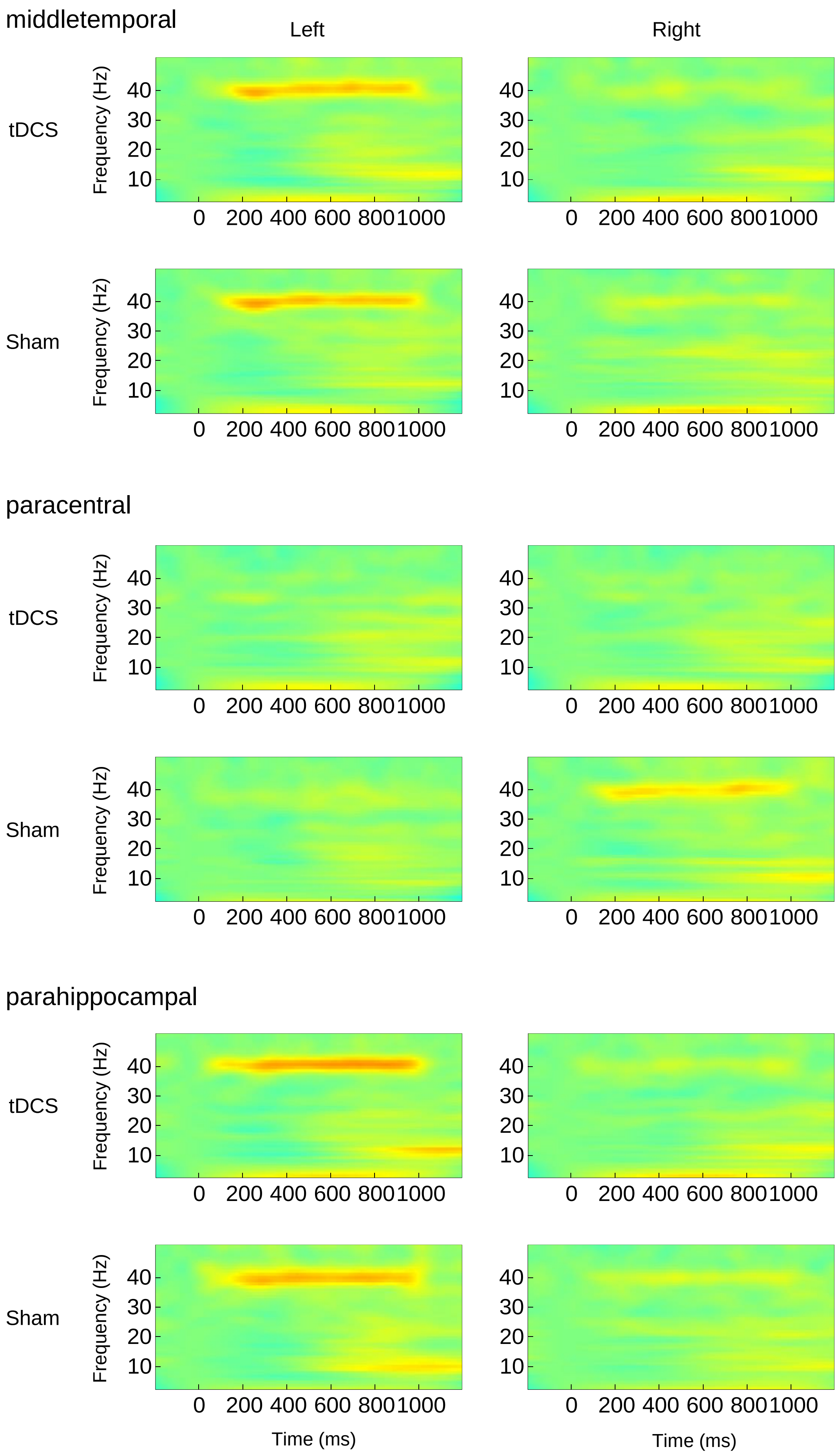
middletemporal
Left
Right
tDCS
Sham
paracentral
parahippocampal
Frequency (Hz)
40
30
20
10
0
200
400
600
800
1000
Time (ms)

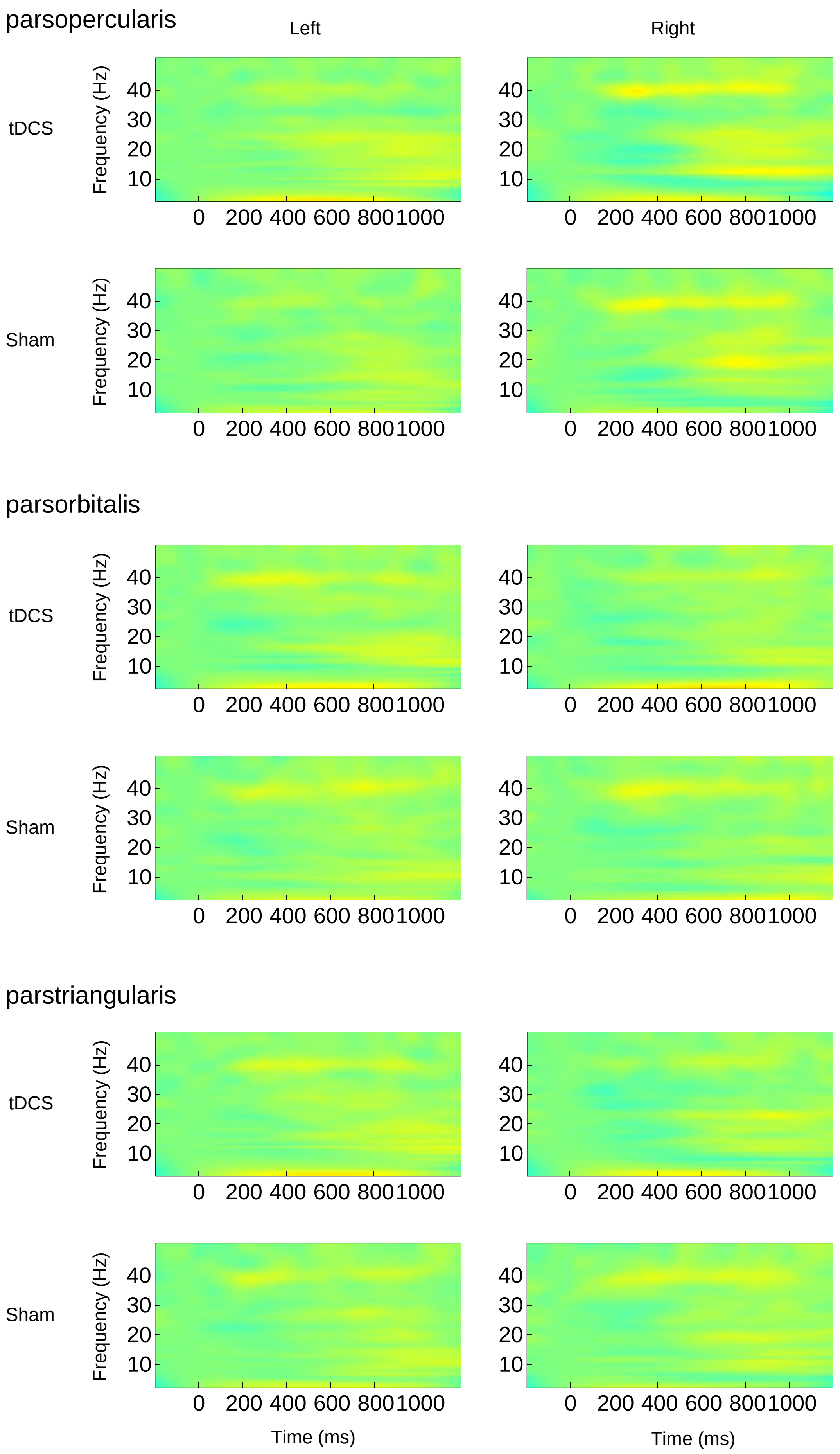
parsopercularis
Left
Right
tDCS
Sham
parsorbitalis
tDCS
Sham
parstriangularis
tDCS
Sham
Frequency (Hz)
10
20
30
40
0
200
400
600
800
1000
Time (ms)

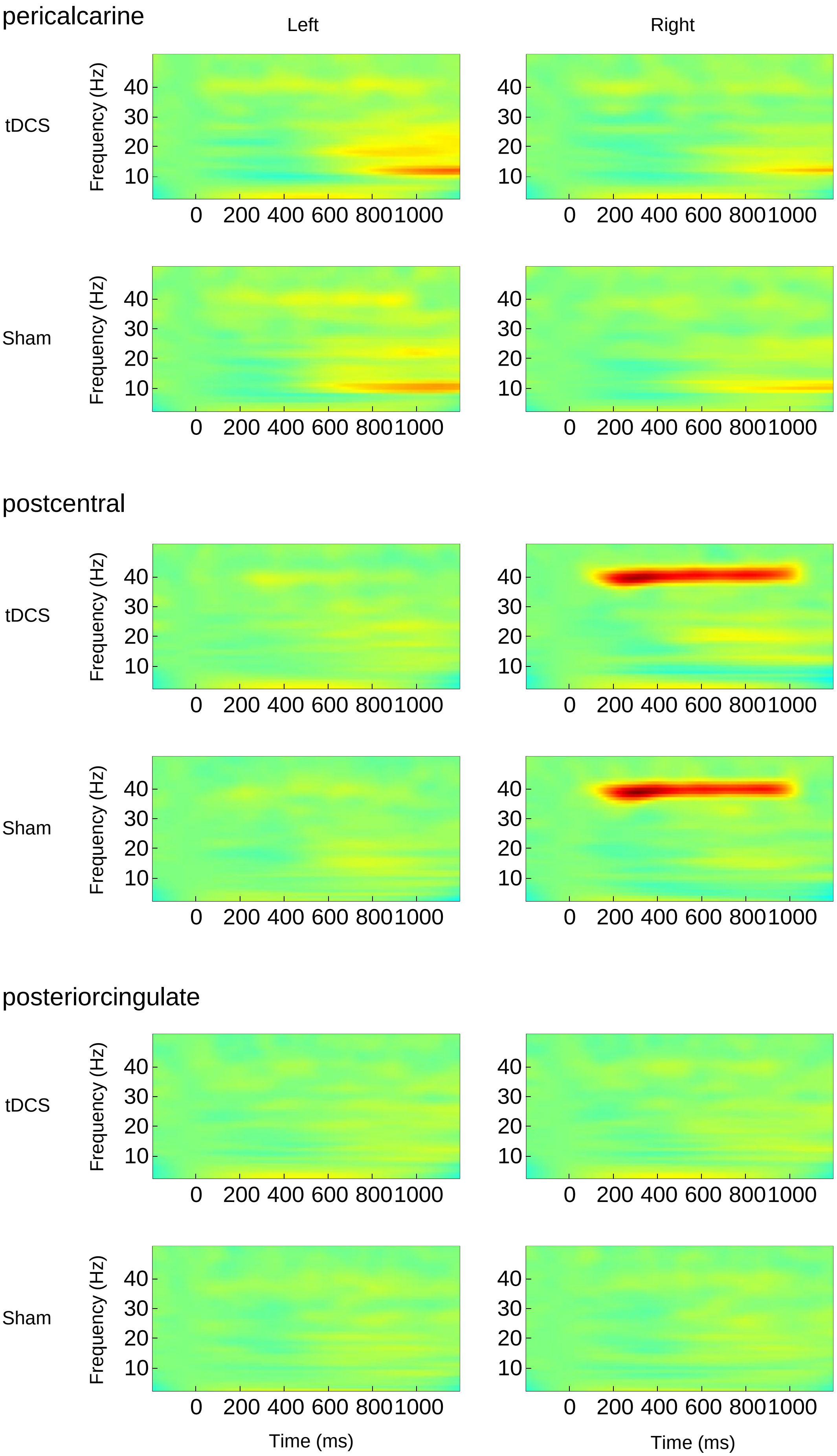
pericalcarine
Left
Right
tDCS
Sham
Frequency (Hz)
10
20
30
40
0
200
400
600
800
1000
postcentral
posteriorcingulate
Time (ms)

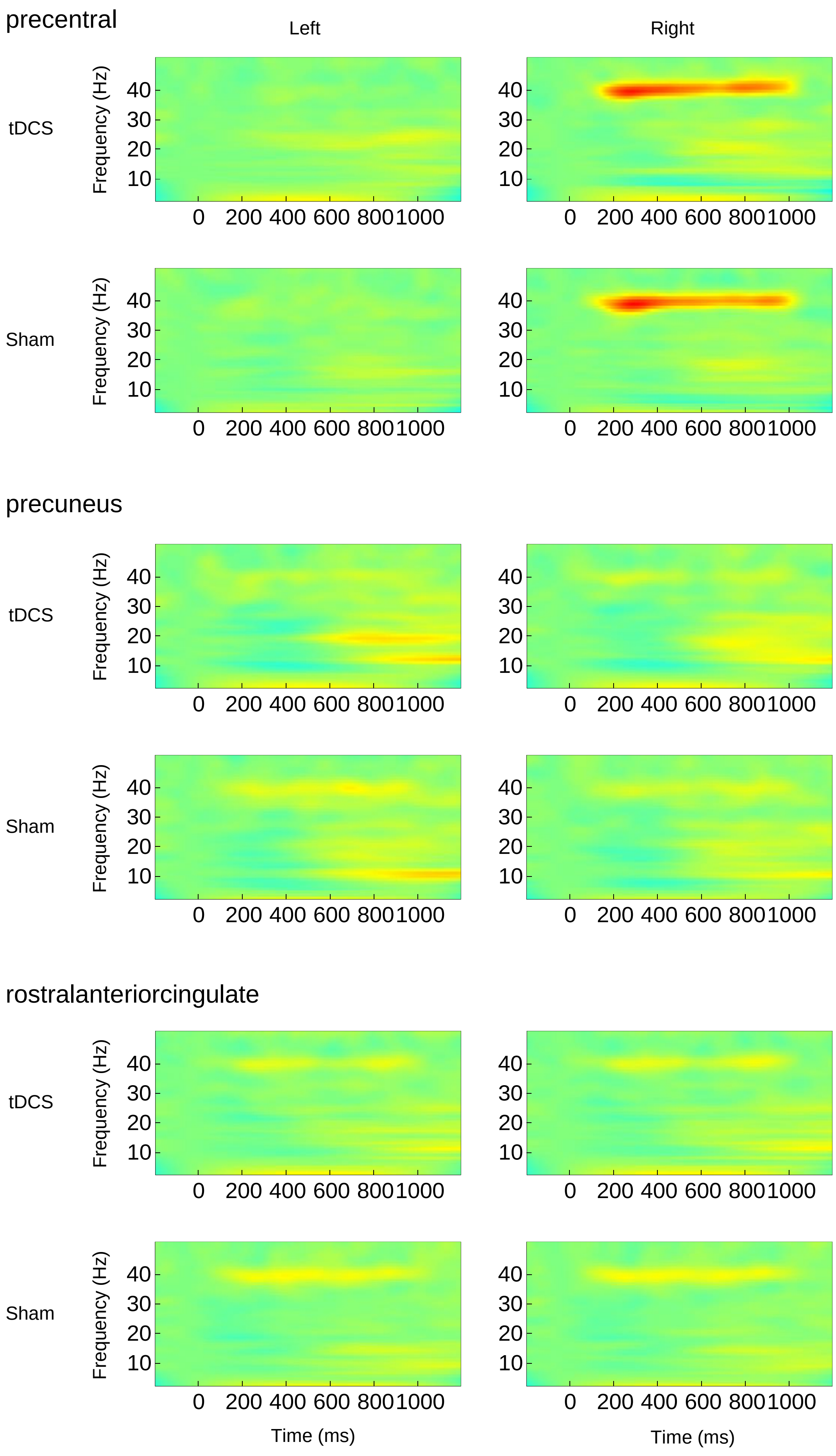
precentral
Left
Right
tDCS
Sham
Frequency (Hz)
40
30
20
10
0 200 400 600 800 1000
precuneus
tDCS
Sham
rostralanteriorcingulate
tDCS
Sham
Time (ms)
Time (ms)

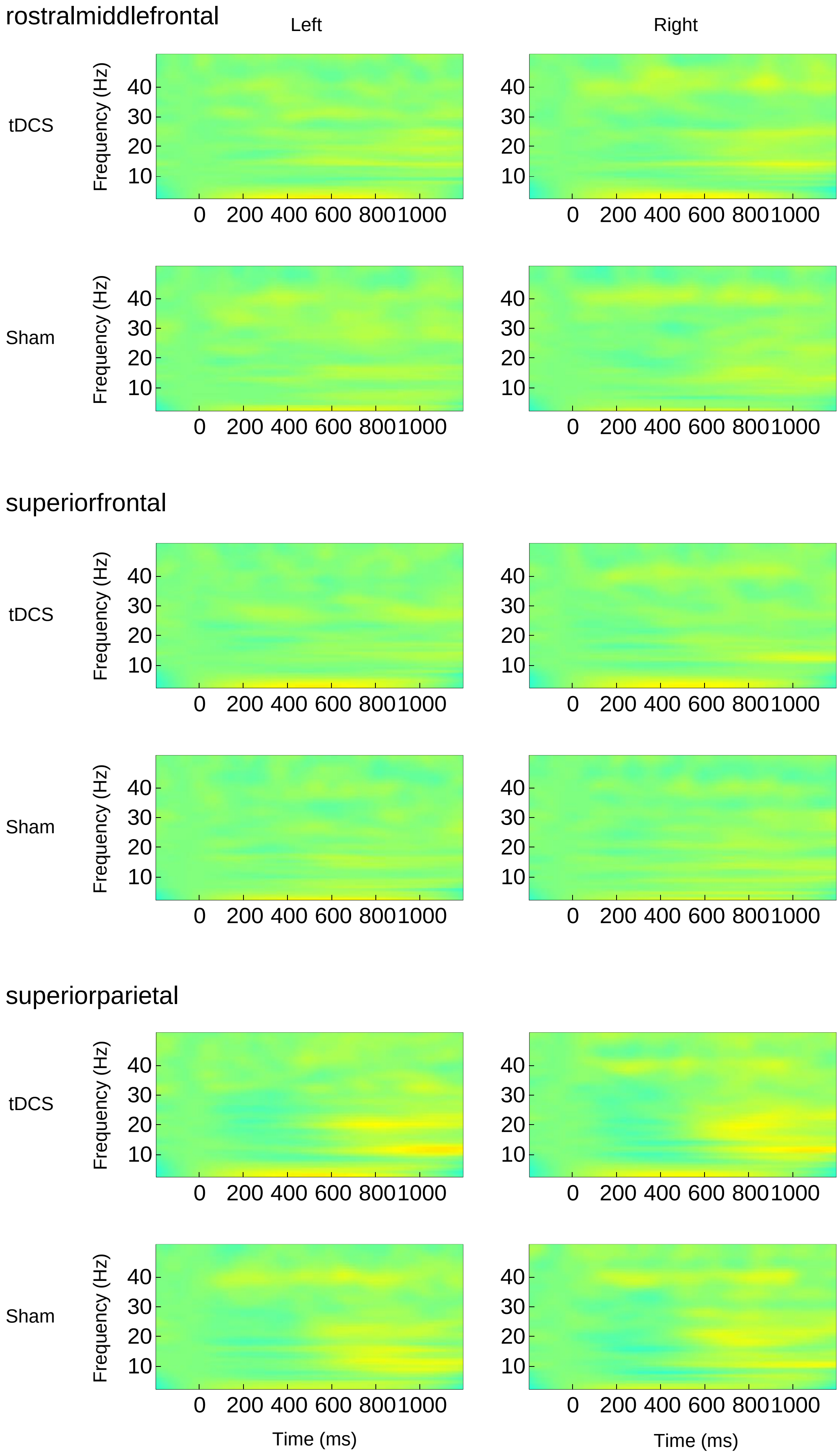
rostralmiddlefrontal
Left
Right
tDCS
Sham
superiorfrontal
tDCS
Sham
superiorparietal
tDCS
Sham
Frequency (Hz)
10 20 30 40
0 200 400 600 800 1000
Time (ms)
Time (ms)

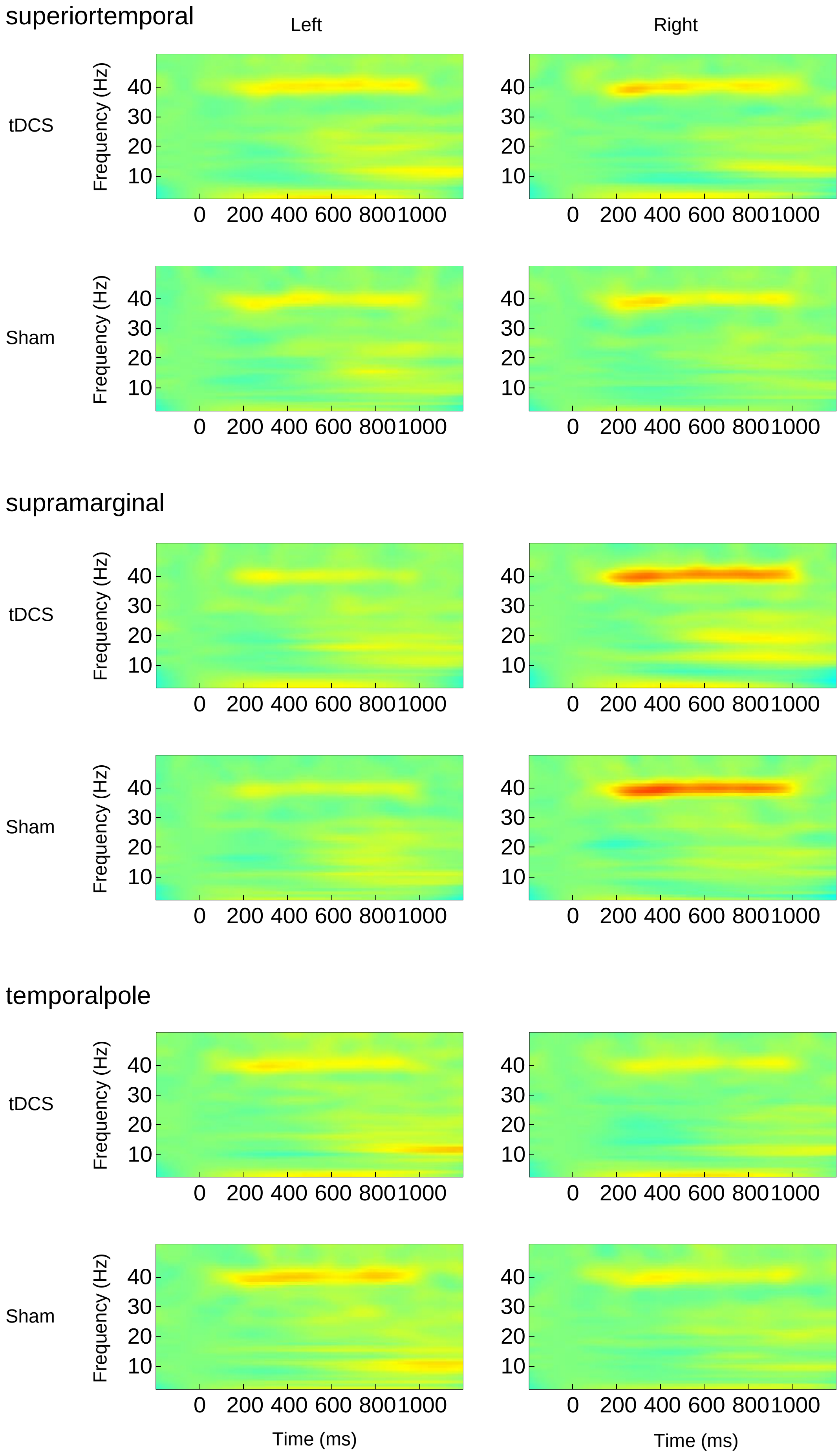
superiortemporal
Left
Right
tDCS
Sham
supramarginal
tDCS
Sham
temporalpole
tDCS
Sham
Frequency (Hz)
10
20
30
40
0
200
400
600
800
1000
Time (ms)

transversetemporal

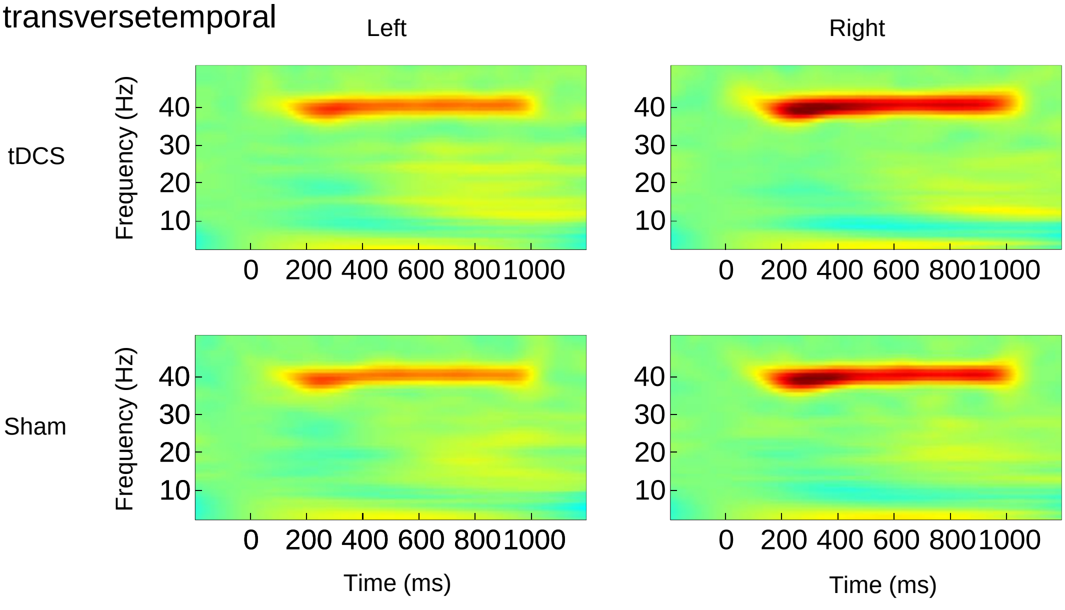

Supplement: S1 Fig — In each map, the x-axis indicates time (ms), and the y-axis indicates frequency (Hz). The color indicates the ITPCs at each time-frequency point. The ITPC peak in the gamma-band (40 Hz) was clearly observed during the 40 Hz auditory stimulation. (PDF) [file pone.0193422.s001.pdf]
