# Supplementary material for: Gamma-band auditory steady-state response after frontal tDCS: A double-blind, randomized, crossover study
Source: PLoS One. 2018 Feb 28;13(2):e0193422. doi: 10.1371/journal.pone.0193422 (PMC5830999; doi:10.1371/journal.pone.0193422)
Supplement: S2 Fig — In each map, the x-axis indicates time (ms), and the y-axis indicates frequency (Hz). The color indicates the ITPCs at each time-frequency point. The ITPC peak in the gamma-band (40 Hz) was clearly observed during the 40 Hz auditory stimulation. (PDF) [file pone.0193422.s002.pdf]

# ITPC

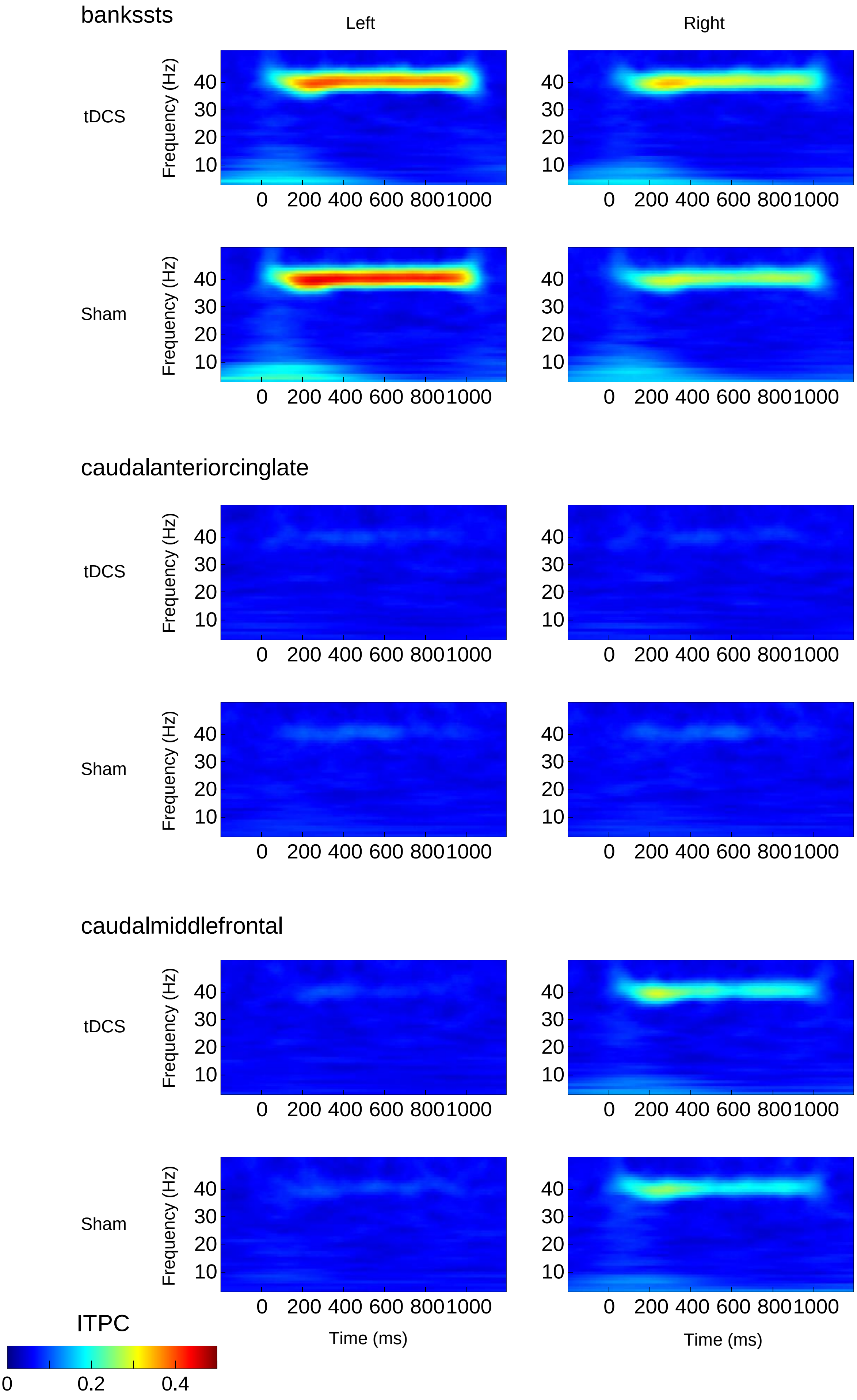
bankssts
Left
Right
tDCS
Sham
Frequency (Hz)
40
30
20
10
0 200 400 600 800 1000
caudalanteriorcinglate
caudalmiddlefrontal
Time (ms)
ITPC
0
0.2
0.4

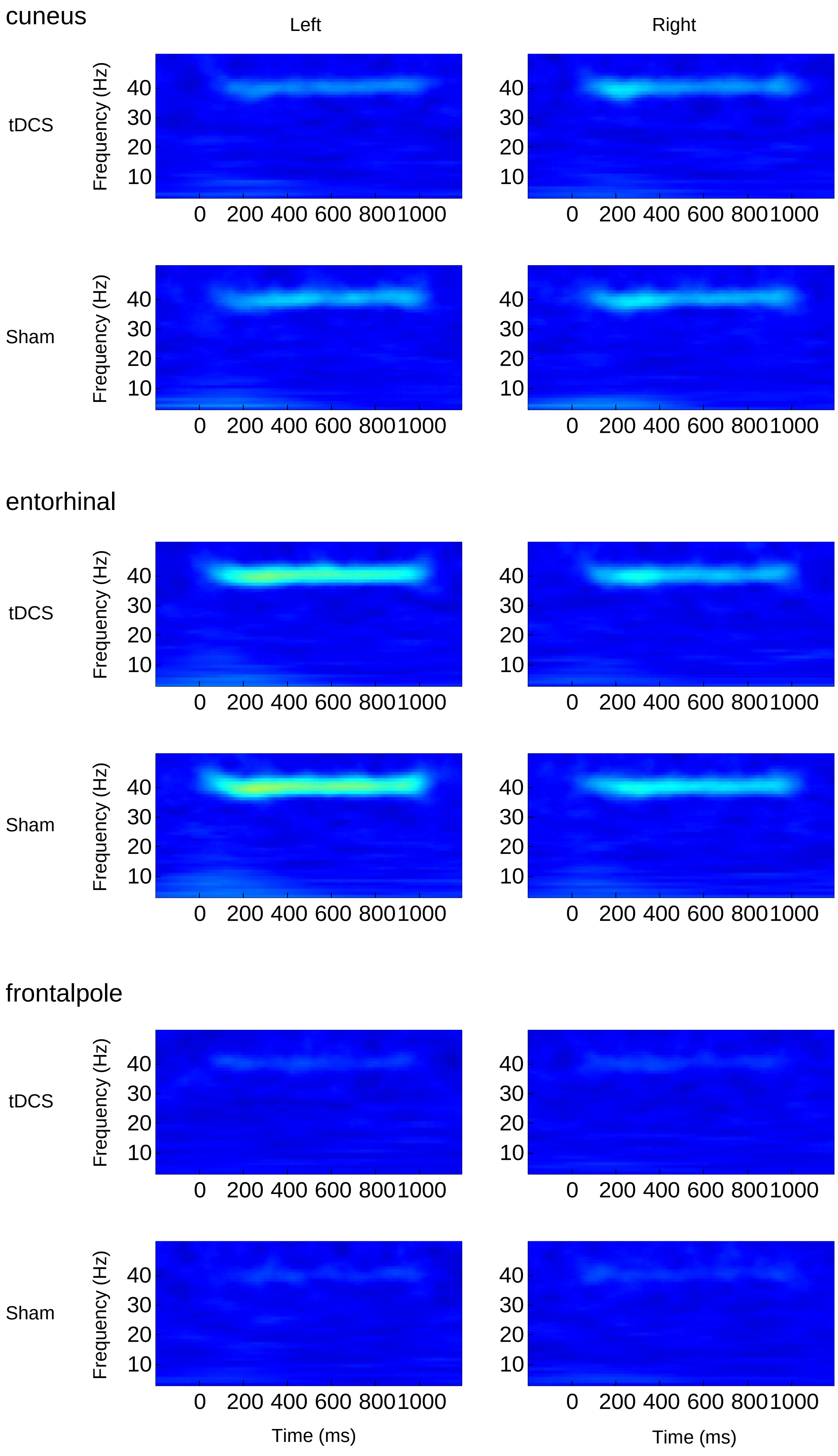
cuneus
Left
Right
tDCS
Sham
entorhinal
frontalpole
Frequency (Hz)
40
30
20
10
0
200
400
600
800
1000
Time (ms)

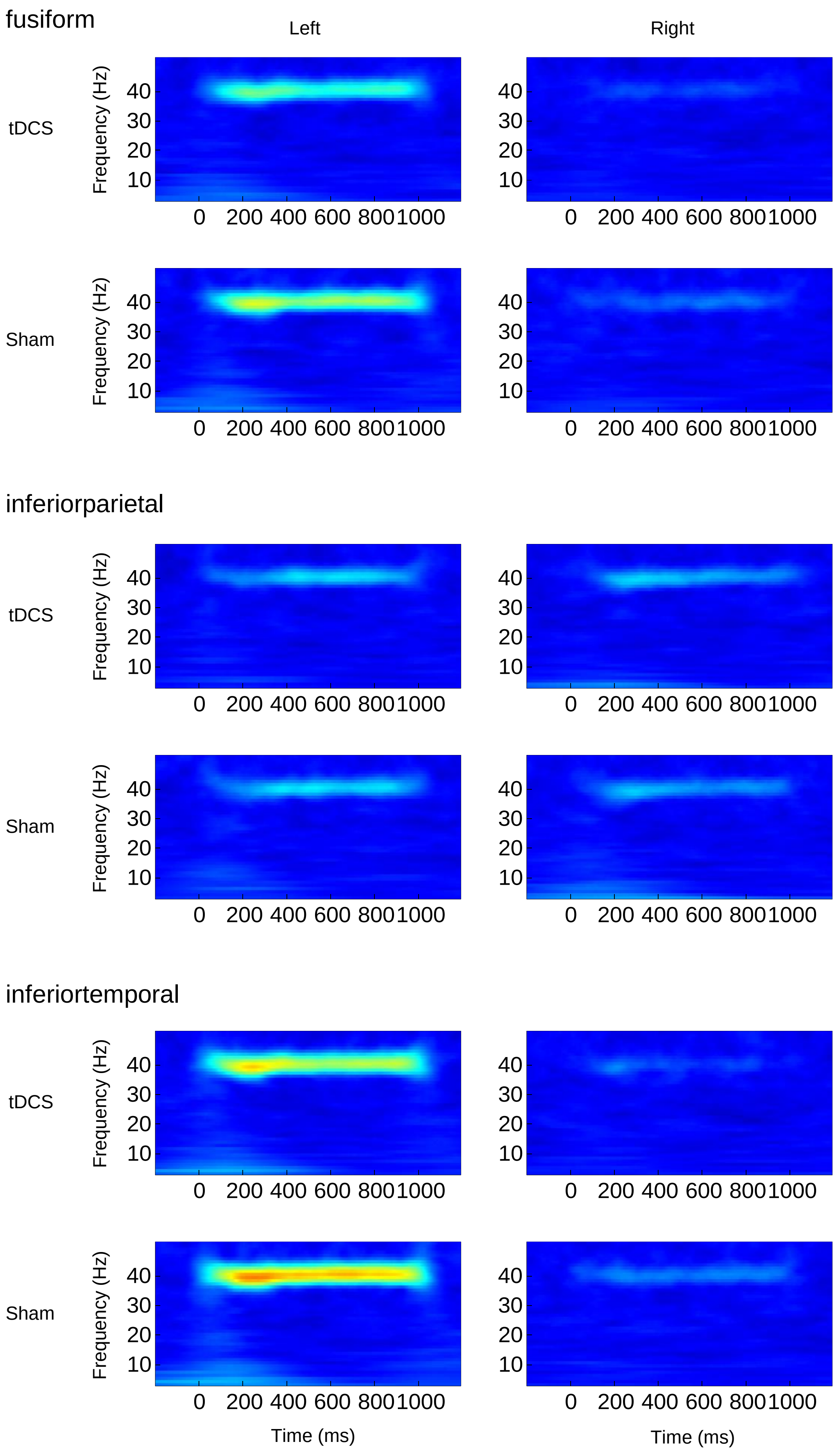
fusiform
Left
Right
tDCS
Sham
Frequency (Hz)
40
30
20
10
0
200
400
600
800
1000
inferiorparietal
inferiortemporal
Time (ms)

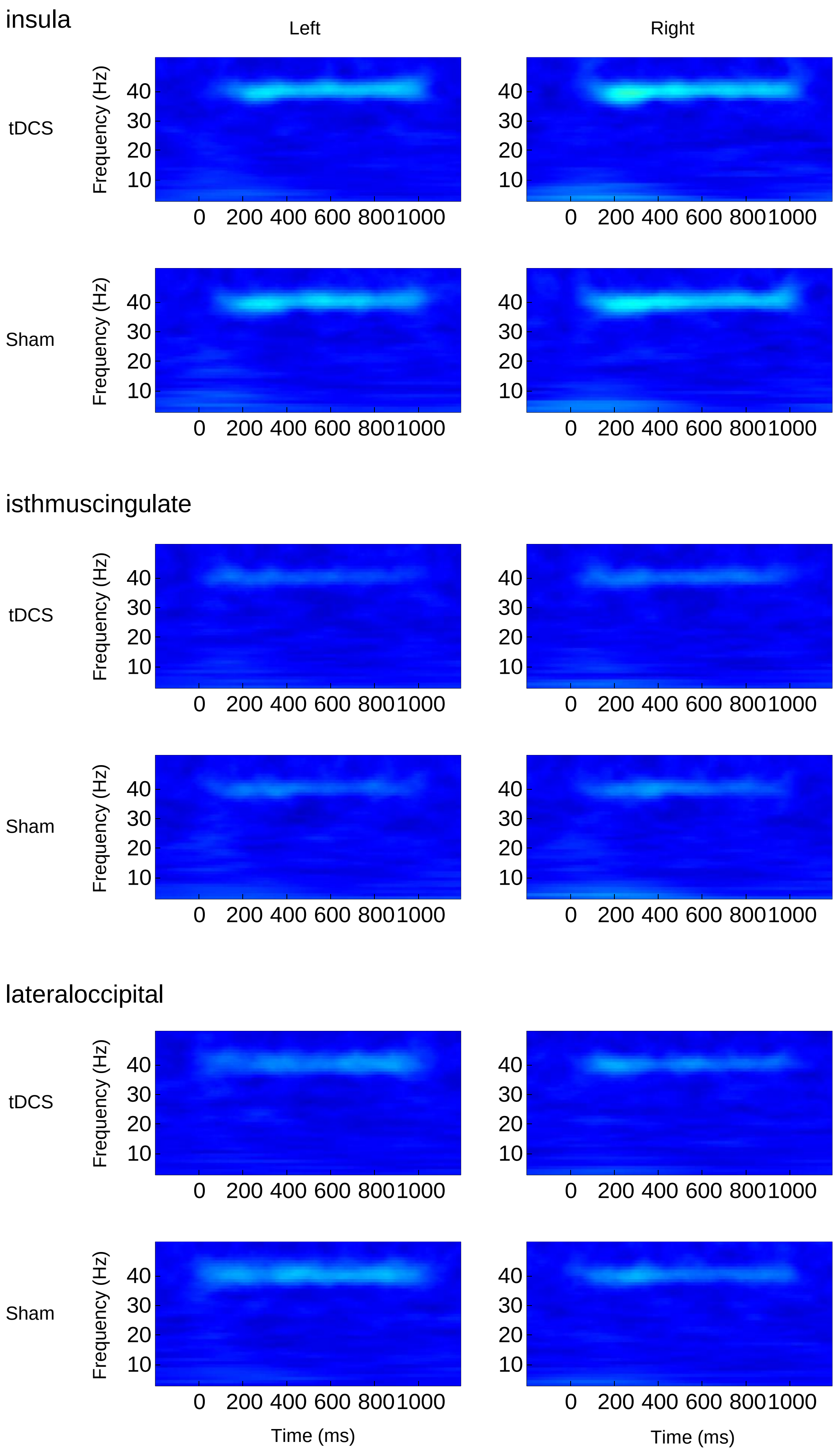
insula
Left
Right
tDCS
Sham
isthmuscingulate
lateraloccipital
Frequency (Hz)
40
30
20
10
0
200
400
600
800
1000
Time (ms)

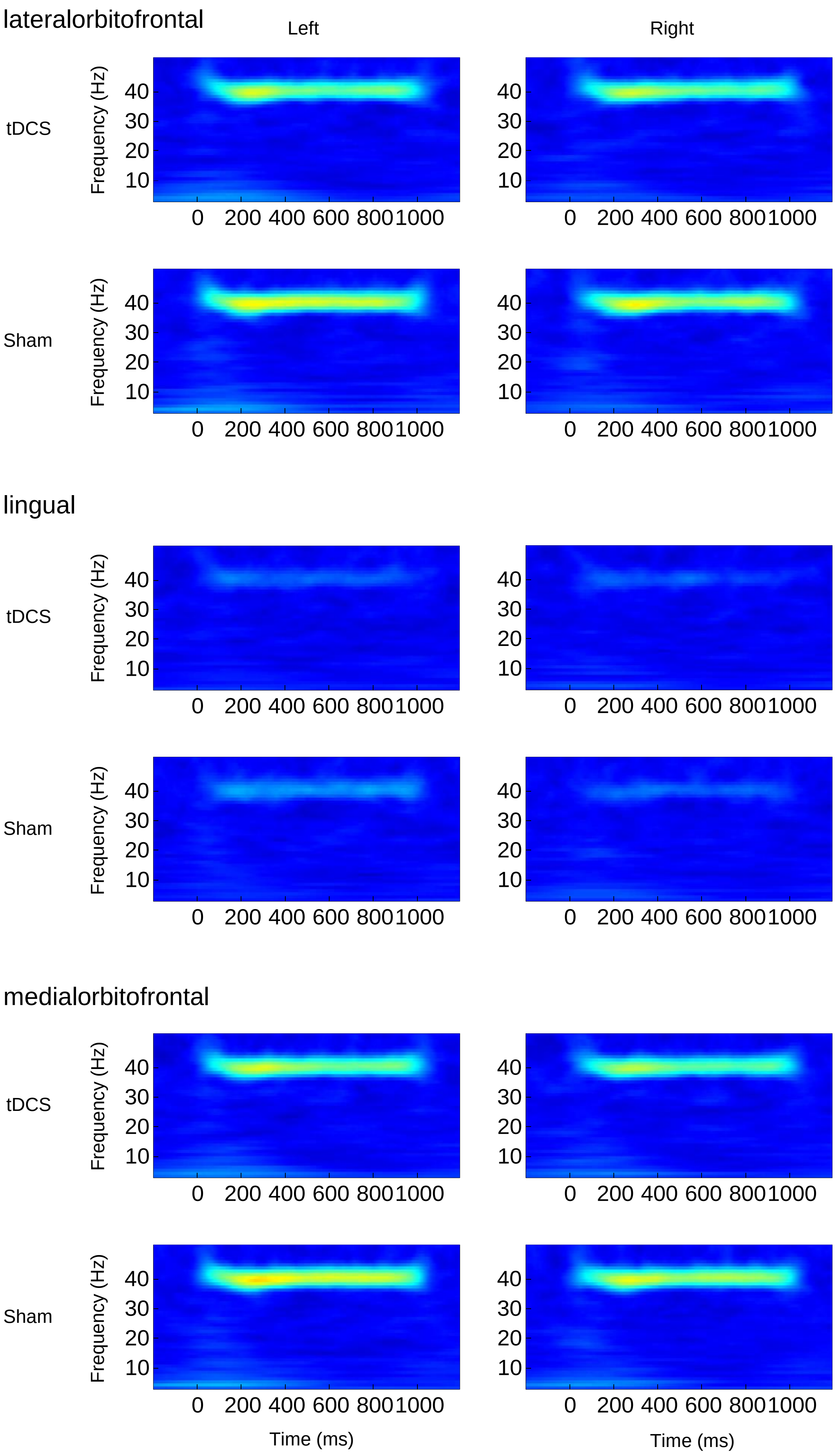
lateralorbitofrontal
Left
Right
tDCS
Sham
lingual
medialorbitofrontal
Frequency (Hz)
10 20 30 40
0 200 400 600 800 1000
Time (ms)

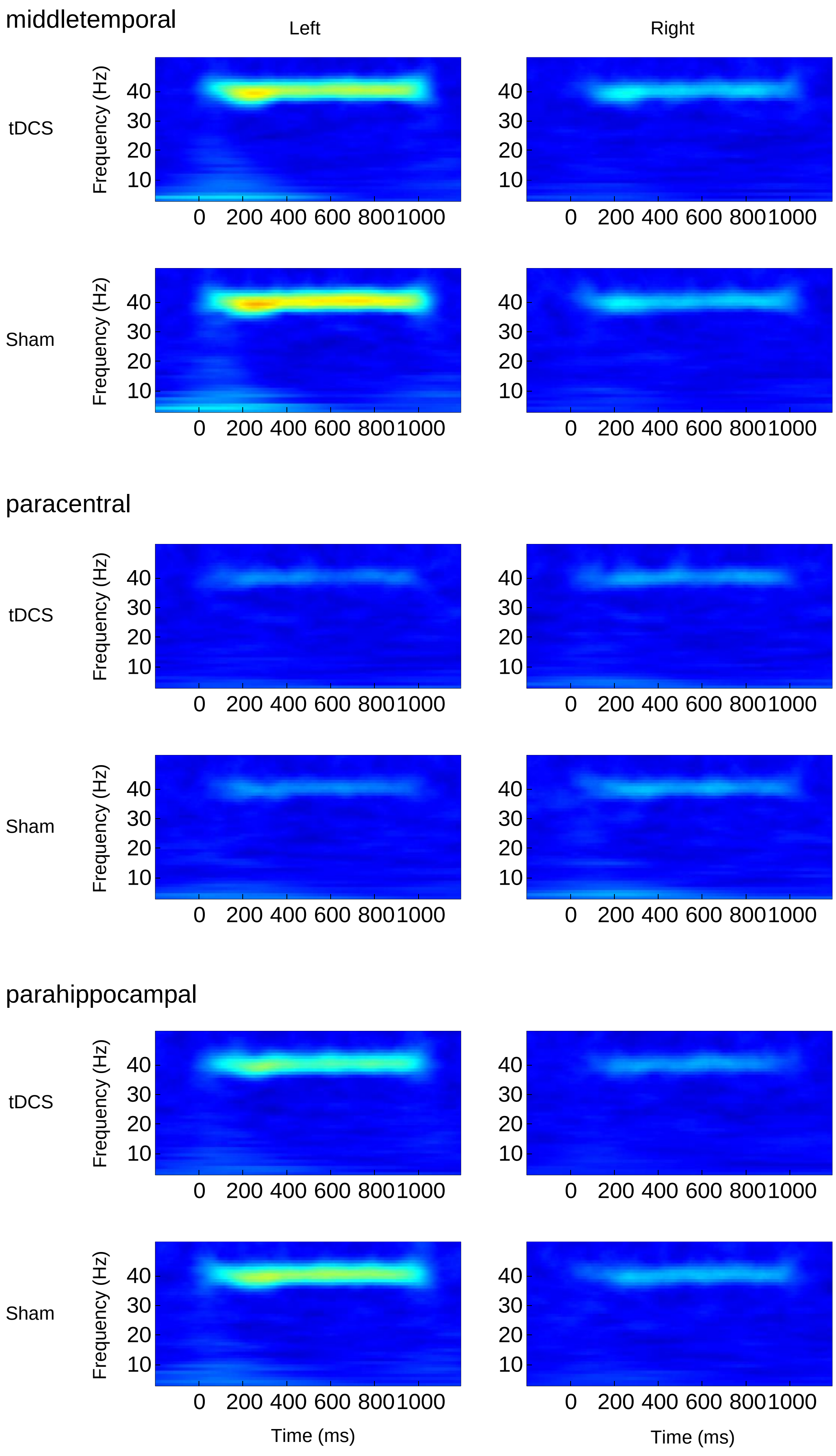
middletemporal
Left
Right
tDCS
Sham
paracentral
tDCS
Sham
parahippocampal
tDCS
Sham
Frequency (Hz)
10
20
30
40
0
200
400
600
800
1000
Time (ms)

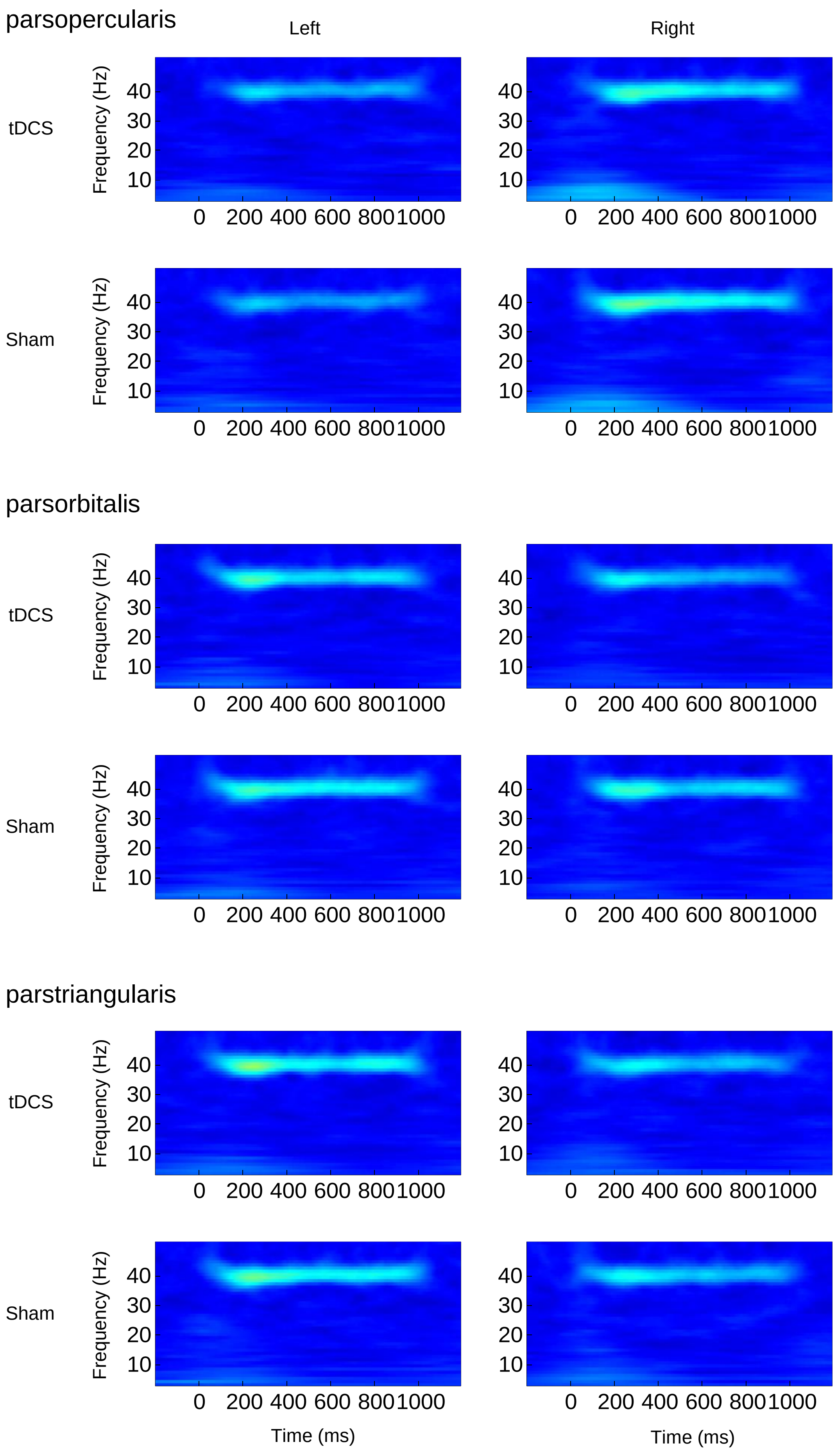
parsopercularis
Left
Right
tDCS
Sham
Frequency (Hz)
40
30
20
10
0
200
400
600
800
1000
parsorbitalis
parstriangularis
Time (ms)

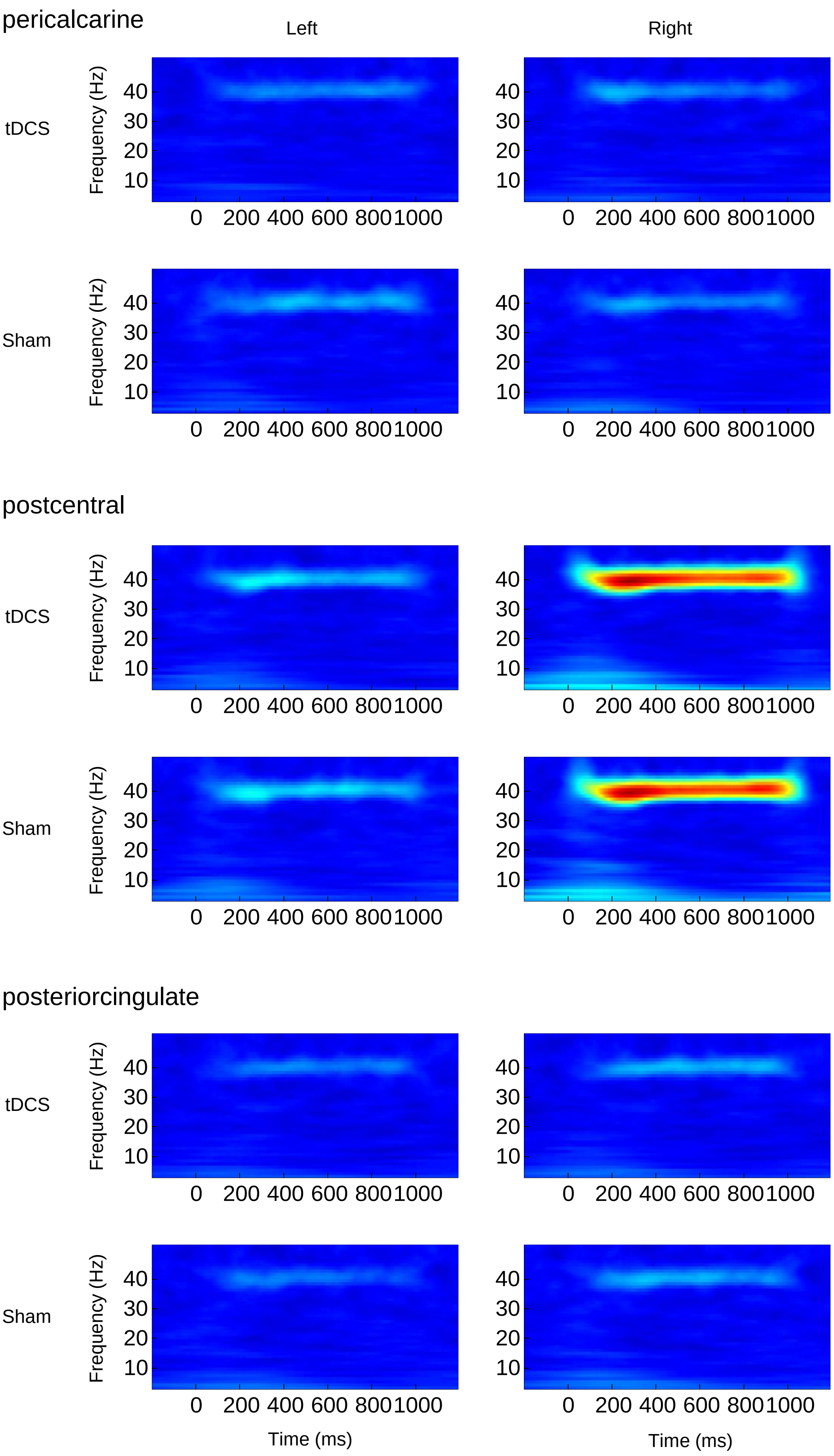
pericalcarine
Left
Right
tDCS
Sham
Frequency (Hz)
40
30
20
10
0
200
400
600
800
1000
postcentral
posteriorcingulate
Time (ms)

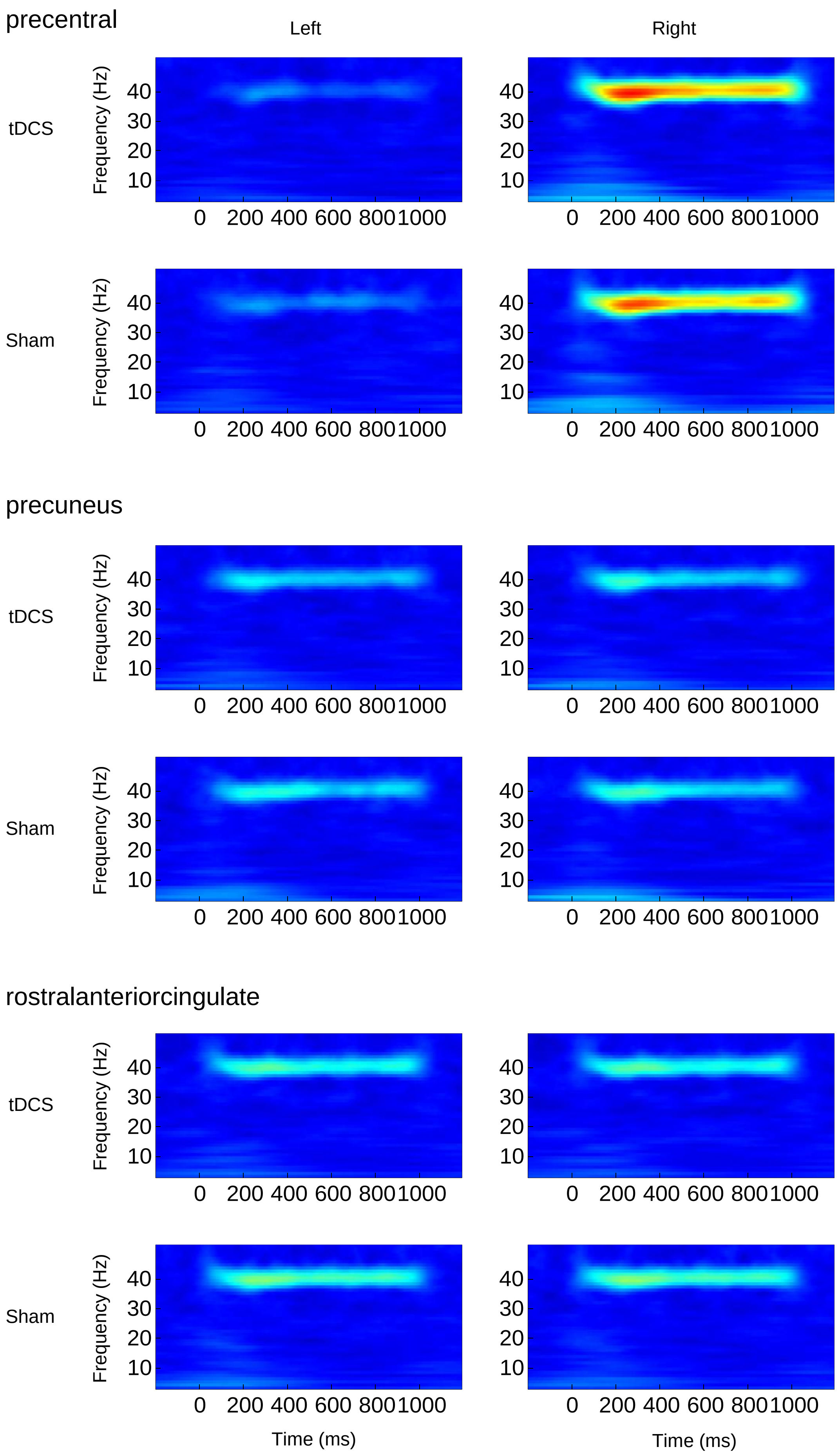
precentral
Left
Right
tDCS
Sham
Frequency (Hz)
10
20
30
40
0
200
400
600
800
1000
precuneus
rostralanteriorcingulate
Time (ms)

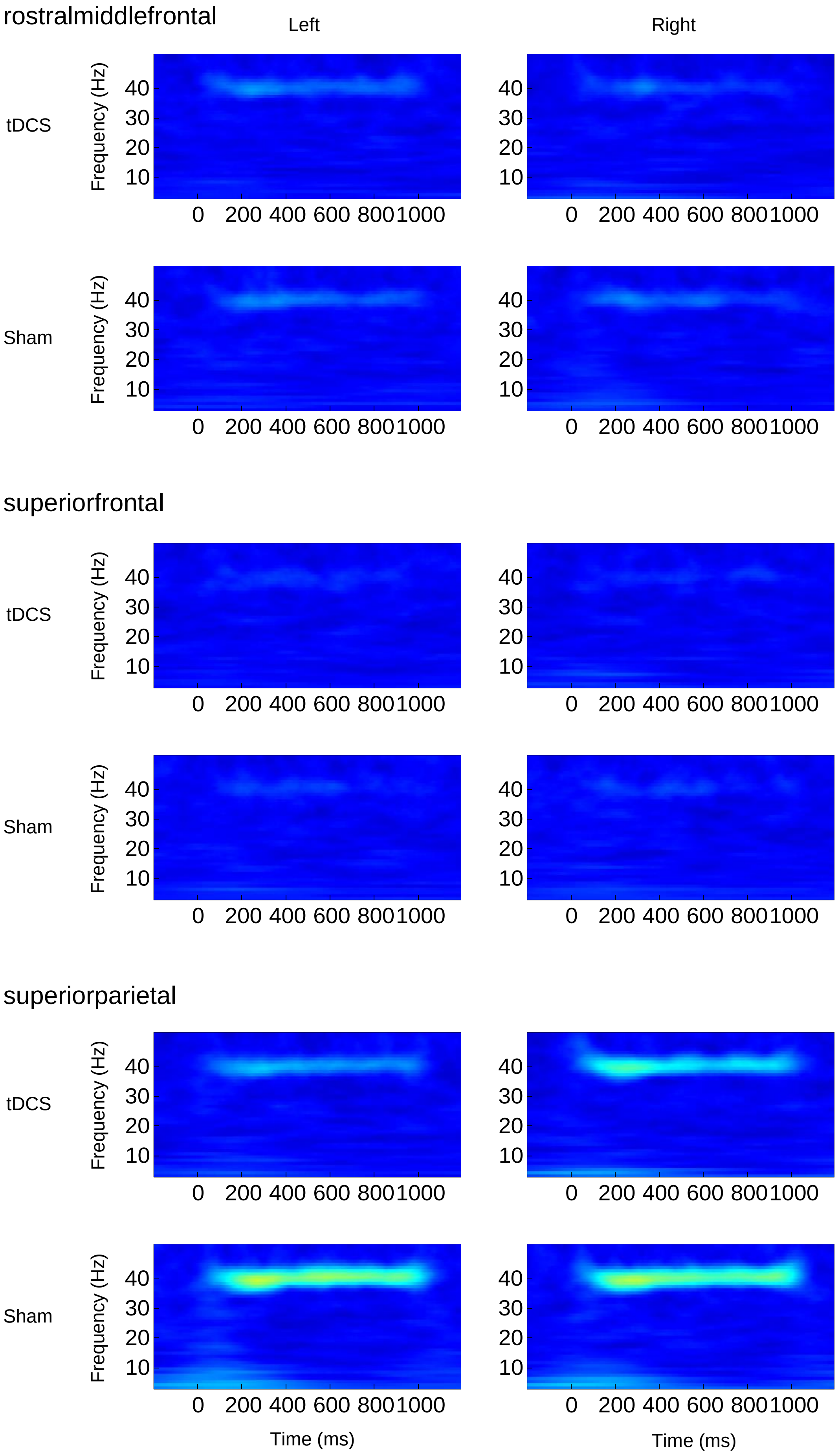
rostralmiddlefrontal
Left
Right
tDCS
Sham
Frequency (Hz)
10
20
30
40
0
200
400
600
800
1000
superiorfrontal
superiorparietal
Time (ms)

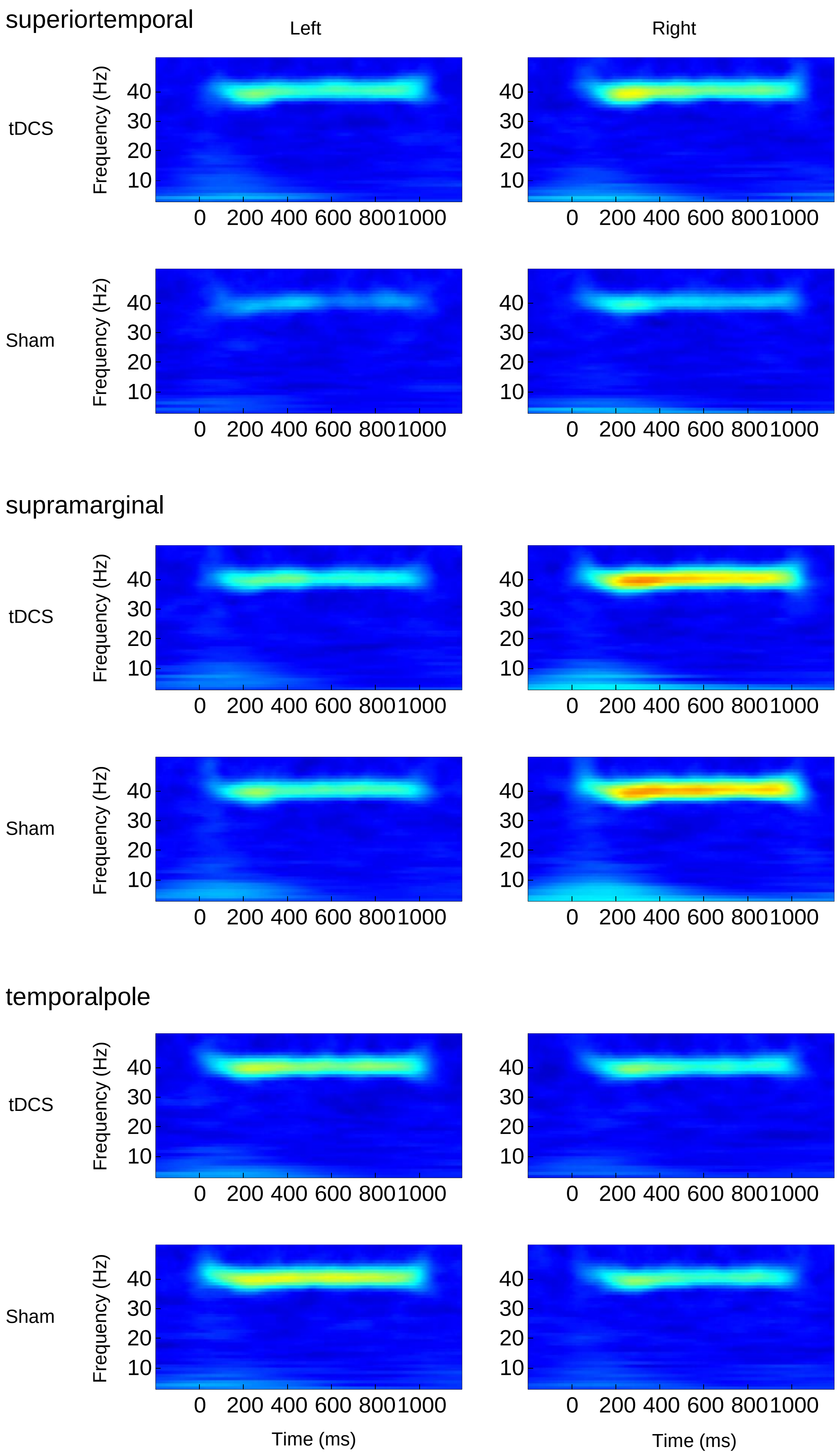
superiortemporal
Left
Right
tDCS
Sham
supramarginal
tDCS
Sham
temporalpole
tDCS
Sham
Frequency (Hz)
10
20
30
40
0
200
400
600
800
1000
Time (ms)

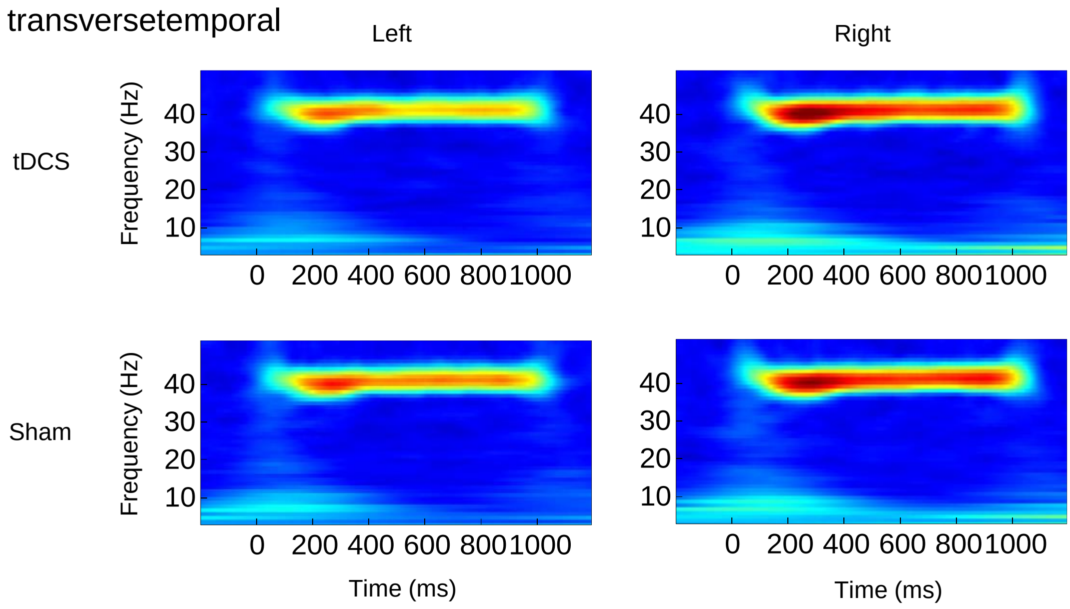
transversetemporal
Left
Right
tDCS
Sham
Frequency (Hz)
40
30
20
10
0
200
400
600
800
1000
Time (ms)
